# Evaluation of the Anti-Aging Effects of a Probiotic Combination Isolated From Centenarians in a SAMP8 Mouse Model

**DOI:** 10.3389/fimmu.2021.792746

**Published:** 2021-12-02

**Authors:** Xin Fang, Mengyun Yue, Jing Wei, Yun Wang, Daojun Hong, Bo Wang, Xiaoting Zhou, Tingtao Chen

**Affiliations:** ^1^ Department of Neurology, The First Affiliated Hospital of Nanchang University, Nanchang, China; ^2^ National Engineering Research Center for Bioengineering Drugs and the Technologies, Institute of Translational Medicine, Nanchang University, Nanchang, China

**Keywords:** aging, SAMP8 mice, TLR4/NFκB, neuroinflammation, probiotic combination

## Abstract

Population aging is a prominent global problem in today’s society. However, there are currently no good methods to treat or prevent aging, so anti-aging research has crucial implications. In this research, we screened bacteria from centenarians, and finally selected four probiotics *(Lactobacillus fermentum SX-0718, L. casei SX-1107, Bifidobacterium longum SX-1326*, and *B. animalis SX-0582*) to form a probiotic combination. By using the senescence accelerated mouse prone 8 (SAMP8) model, the anti-aging effects of the probiotic combination were evaluated by using behavioural testing, neuroinflammation, intestinal inflammation, and intestinal microbiota. The results showed that probiotic combination improved the impaired spatial memory, motor dysfunction, and decreased exploratory behavior in aging mice. The probiotic combination inhibited Toll-like receptor 4 (TLR4)/nuclear factor kappa B (NFκB)-induced neuroinflammation and up-regulated the expression of Sirt 1 to protect hippocampal neurons. At the same time, the probiotic combination regulated the intestinal microbiota, reduced the relative abundance of *Alistipes* and *Prevotella* in SAMP8 mice, inhibited TLR4/NFκB-induced intestinal inflammation, and increased the expression of intestinal permeability related proteins zonula occludens-1 (ZO-1) and Occuldin. The anti-aging effects of the probiotic combination may be through the regulating intestinal microbiota and inhibiting TLR4/NFκB-induced inflammation. This research provides the basis and technical support for the future production and application of the probiotic combination.

## Introduction

Aging is a process that almost all living organisms go through, characterised by the gradual decline of in the body’s cell, tissue, and organ functions over time, as well as reduced cognitive and memory functions (1). Globally, the population over the age of 65 is 617 million (8.5%), and this number may reach 1.6 billion by 2050 (2). With the aging of China’s population, the incidence of aging-related diseases - Parkinson’s disease (PD), Alzheimer’s disease (AD), malignant tumours, and others – continues to rise, causing a heavy financial burden on the country and the families of patients (3, 4). Although many drugs such as metformin, resveratrol, and rapamycin have been proven to have anti-aging effects, they have not been promoted widely due to high cost, difficulty in extraction, and serious side effects (5). Therefore, finding or developing anti-aging active substances and exploring their mechanisms of action has become a current research hotspot in response to the reality of population aging.

In recent years, inflammatory aging has become a new topic in aging research (6). These studies have shown that inflammatory aging is closely related to the occurrence and development of many senile diseases such as AD, atherosclerosis, PD and osteoporosis (7). Inflammatory aging refers to the chronic and progressive increase in the pro-inflammatory state of the body during the natural aging process. The main reason is the imbalance between pro-inflammatory and anti-inflammatory cytokines in the body, which ultimately leads to an increase in the pro-inflammatory response (8). Pro-inflammatory cytokines can induce stem cell senescence, which is the cellular basis for the aging of tissues and organs. Senescent cells secrete cytokines, growth factors, proteases, and other substances that cause inflammation and destroy the cellular microenvironment, leading to reduced cell survival. It affects the proliferation and differentiation of cells and induces stem cell senescence and aging-related diseases (9). Research has shown that in the elderly, increased levels of serum inflammatory factors such as tumour necrosis factor-α (TNF-α), interleukin-6 (IL-6) and C-reactive protein (CRP) are considered to be risk factors of cardiovascular and degenerative diseases (10). Bruunsgaard et al. (11) found that the increased of TNF-α is correlated positively with the all-cause mortality of elderly men in a follow-up study of 333 relatively healthy elderly people over 80 years of age, indicating that TNF-α has a certain predictive effect on death.

According to recent research reports that there is an important relationship the between intestinal microbiota and inflammatory aging, Inflammation may be caused by decreased in the autoimmune tolerance and the composition of the intestinal microbiota caused by aging, leading to its abnormal immune activation (12). Increased release of gram-negative bacteria and lipopolysaccharide in the intestine leads to chronic inflammation throughout the body, which in turn induces the occurrence of various neurological diseases such as AD, PD, and amyotrophic lateral sclerosis (ALS) (12). The intestinal microbiota can also regulate host behaviour through the brain-gut axis, affect the host’s blood-brain barrier function and basic neurodevelopmental processes such as the maturation of microglia, and participate in the regulation of brain functions (13). The intestinal microbiota participates in the process of aging mainly through regulating oxidative stress, the immune response, and metabolism (13, 14). Aging people have disorders of the intestinal microbiota. Studies have compared the fecal microbes of patients with progeria with their respective healthy siblings. There is a marked decline in the relative abundance of the family *Ruminococcaceae* in patients with progeria, while the families *Erysipelotrichaceaceae* and *Lachnospiraceae* are enriched; these findings are consistent with the mouse model of progeria (15). When the researchers transplanted the intestinal microbiota of normal mice into progeria mice, the average lifespan increased (15). These results indicate that regulating and maintaining the balance of the intestinal microbiota of the elderly may be a means of preventing and treating aging-related diseases and delaying aging. Hence, this topic deserves further discussion and research.

As active microorganisms that are beneficial to human body, probiotics play a great part in maintaining the balance of microorganisms in the intestinal tract. Supplementing probiotics can facilitate the production of immunologically active factors and different types of immunoglobulins by regulating cellular and humoral immunity, participating in inflammation, improving the immune response, and promoting the proliferation of spleen cells (16). Therefore, our group screened probiotics from the faeces of seven centenarians of the Centenarian Village in Ganzhou, Jiangxi province, China. Based on this screening, we chose *Lactobacillus fermentum* SX-0718, *L. casei* SX-1107, *Bifidobacterium longum* SX-1326, and *B. animalis* SX-0582 to prepare a probiotic combination. Then we evaluated the anti-aging effects of this probiotic combination by using the senescence accelerated mouse prone 8 (SAMP8) mice model. Our findings provide a basis for the development of an anti-aging probiotic dietary supplement for elderly people.

## Materials and Methods

### *In Vitro* Experiments

#### Bacterial Strains

The research team screened the faeces of seven centenarians of the Centenarian Village in Ganzhou, Jiangxi province, China; their ages were 103, 107, 102, 105, 100, 101, and 100, respectively. First, the faecal microorganisms were extracted and subjected to serial dilutions. The various dilutions were spread aseptically on the selected culture medium and cultivated in aerobic and anaerobic environments for 24–48 h. According to the colony shape, size, colour, edge, gloss, and texture, 20–40 single colonies were picked and then activated and cultured on the corresponding liquid medium for 24–48 h. The genomic DNA of the activated bacteria was extracted and then sequenced to identify the types of bacteria by using the NCBI database. A total of more than 1,500 strains were screened, and four probiotics were selected to form a probiotic combination (all from Jiangxi Shanxing Biotechnology Co., Ltd, Nanchang, Jiangxi, PR China): *L. fermentum* SX-0718, L*. casei* SX-1107, *B. longum* SX-1326, and *B. animalis* SX-0582. The bacteria were cultivated in De Man-Rogosa-Sharpe (MRS) medium at 37°C under anaerobic conditions with a bacterial density of 1 × 10^9^ colony-forming units (CFU)/mL.

#### Probiotic Evaluation of Isolates

For the acid resistance test, after activation, bacteria were centrifuged at 4500 g for 10 min at 4°C, and the cell pellet was resuspended in phosphate buffered saline (PBS). The cell suspension was diluted in PBS with different pH (3, 5, 7 and 9) and incubated at 37°C, for 4 h. For the bile salt tolerance test, bacteria were inoculated in MRS medium containing different bile salts concentrations (0.0%-0.5% wt/wt) at 37°C for 4 h. After incubation, all bacteria were counted by the plate number method (17).

For antimicrobial testing, pathogenic microorganisms were selected, including *Salmonella typhimurium* ATCC 13311, *Shigella flexneri* ATCC 12022, *Propionibacterium acnes* ATCC 11827, *Sh. dysenteriae* 301, *Enterohemorrhagic coli* O157, *S. enteritidis* ATCC 13076, *Listeria monocytogenes* ATCC 19111, *Staphylococcus aureus Cowan1* and *Candida albicans* SC531. They were cultured overnight and spread on the lysosomal broth (LB) (Hopbio, Hb0384-1, Qingdao) agar plate surface. Then, an Oxford cup was placed on the surface of the agar, and the bacterial supernatant (200 μL) was added. The size of the inhibition zone around the Oxford Cup was measured (18).

### Experimental Design and Processing

The mice used in this experiment – SAMP8, a rapidly aging mouse model, and SAMR1, the corresponding normal aging model – were purchased from the Peking University Health Science Center. The 3-month-old male mice were maintained in a standard environment for 2 weeks before beginning the experiments. The standard environment for mouse breeding comprised a 12-h photoperiod, a temperature of 22 ± 3°C, relative humidity of 50% ± 15%, and free access to food and water. Then, the mice were divided into four groups: (i) the control (C) group (n = 10), SAMR1 mice, daily gavage of normal saline (the same volume as used for the probiotic combination); (ii) the model (M) group (n = 9), daily gavage of normal saline daily (the same volume as used for the probiotic combination); (iii) the low-dose probiotic (L) group (n = 10), daily gavage of 1 × 10^7^ CFU/mL *L. fermentum* SX-0718 + 1 × 10^7^ CFU/mL *L. casei* SX-1107 + 1 × 10^7^ CFU/mL *B. longum* SX-1326 + 1 × 10^7^ CFU/mL *B. animalis* SX-0582 for 18 weeks; (iv) the high-dose probiotic (H) group (n = 10), daily gavage of 1 × 10^9^ CFU/mL *L. fermentum* SX-0718 + 1 × 10^9^ CFU/mL *L. casei* SX-1107 + 1 × 10^9^ CFU/mL *B. longum* SX-1326 + 1 × 10^9^ CFU/mL *B. animalis* SX-0582, for 18 weeks. After the probiotic treatment, all mice underwent behavioural testing. They were then anaesthetised and the brain and colon were collected (**Supplementary Figure 1A**).

### Aging Score

Animals in each group were scored objectively for 11 indicators including fur gloss, fur roughness, the degree of hair loss, skin ulcers, eye damage, corneal turbidity, corneal ulcers, cataracts, kyphosis, reactivity, and the passive escape response. Each index is divided into 4-5 grades, and the score is calibrated. The higher the score an animal gets, the higher its degree of aging (19).

### Behavioural Experiments

The pole test was used to detect motor dysfunction in mice. The apparatus is a metal rod with a diameter of 1 cm and a length of 50 cm. To prevent the mice from slipping, the metal rod was wrapped with bandage gauze, and the bottom of the metal rod was placed in a cage. The mouse was placed face down on the top of the pole, and the latency for the moue to fall freely to the cage (hind limbs touch the bottom of the cage) was recorded. Each mouse was tested three times, 15 min apart, and the average value was calculated.

The open field test was used to evaluate the changes in exploratory behaviour and anxiety of each mouse when exposed to a new environment. The apparatus is a square divided into 25 squares of equal area; the edge area and the central area are defined. The free movement of mice was recorded for 10 min. After each experiment, the experimental area was cleaned to remove any odour left by the previous mouse so that it would not affect the behaviour of the subsequent mouse.

The Barnes maze was used to test the spatial memory ability. The animals were placed individually in the target box of the target hole and allowed to adapt to the apparatus for 2 min the day before the start of the test. On day 1 of testing, a transfer device was used to place the animal in the centre of the maze. Then, each mouse was guided to the target hole, and the animals were permitted to stay in the box for 2 min. Each animal was observed for a maximum of 3 min at a time. During this period, if the animal could not find the target box, it was guided to the target box and allowed to stay there for 2 min. The animals were trained each day for 9 days. On the last day of probe test, count the incubation period of each group of mice, the stay time in the target area, the stay time in the reverse target area, and the correct number of holes.

### Western Blot

One gram of tissue was homogenised in radioimmunoprecipitation assay (RIPA) buffer containing protease inhibitor cocktail and phenylmethylsulphonyl fluoride (PMSF) with electric homogeniser. The sample was centrifuged at 12000 g for 10 min a 4°C. The supernatant was removed and the protein concentration was determined with a BCA protein assay kit. The samples were then subjected to sodium dodecyl sulphate–polyacrylamide gel electrophoresis (SDS-PAGE) to separate protein. The wet transfer method was used to transfer the separated protein to a polyvinylidene fluoride membrane (PVDF). After the transfer, the membrane was blocked and then incubated with the appropriate primary and secondary antibodies according to the manufacturer’s instructions. Finally, the membrane was incubated with a chemiluminescent substrate and the protein bands were visualised with an automatic gel imaging analyser. The primary antibodies used included: rabbit anti-β-actin (β-actin; 1:1000; Cell Signaling Technology; Cat# 4970S), rabbit anti-Bcl-2 associated X Protein (Bax, 1:1000; Cell Signaling Technology, Cat# 14796S), rabbit anti- B-cell lymphoma-2 (Bcl-2, 1:1000; Cell Signaling Technology, Cat# 3498S), rabbit anti- phosphorylated-AKT (p-AKT; 1:1000; Sangon Biotech, Cat# D151499), rabbit anti- AKT (1:1000; Sangon Biotech, Cat# D151621), rabbit anti- silent information regulator 1 (Sirt 1; 1:1000; Cell Signaling Technology, Cat#9475), mouse anti-Toll-like receptor 4 (TLR4; 1:1000; Santa Cruz Biotechnology, Cat# sc-293072), rabbit anti-myeloid differentiation primary response gene 88 (MyD88; 1:1000; Proteintech; Cat# 23230-1-AP), rabbit anti-phosphorylated-p65 (p-p65; 1:1000; Abcam; Cat# ab86299), rabbit anti-p65 (p65 1:1000; Cell Signaling Technology; Cat# 8242S), rabbit anti-tight junction protein 1 (zona occludens 1, ZO-1; 1:5000; Proteintech; Cat# 21773-1-AP), and rabbit anti-Occludin (Occludin 1:1000; Proteintech; Cat# 13409-1-AP).

### Immunofluorescence and Inflammatory Factor Detection

Brains were fixed in paraformaldehyde and then subjected to dehydration, clearing, wax immersion, and embedding. The embedded tissue was sliced continuously at a thickness of 5 μm. The sections were deparaffinised and equilibrated to water, subjected to antigen retrieval, and incubated in 5% bovine serum albumin (BSA) for 30 min. The sections were then incubated with the appropriate primary and secondary antibodies. The nuclei were then stained with DAPI. After mounting the sections, they were viewed under an upright fluorescence microscope and images were captured. The number of NeuN-positive cells was counted by using the ImageJ software (National Institutes of Health). The average cell number/field of view was used for statistical analysis.

To detect inflammatory factors, protein was extracted from the brainss following the same method described for western blot. The following kits were used to evaluate the levels of inflammatory factors in the hippocampus of mice, following the manufacturer’s instructions: IL-1β (Proteintech, Cat# KE10003), TNF-α (Proteintech, Cat# KE10002) and IL-6 (Proteintech, Cat# KE10007).

### Bacterial DNA Extraction

For microbiota analysis, the faeces of each group of mice (n=8) were collected. A genomic DNA kit (Qiagen, Cat#51804) was used to extract fecal genomic DNA, following the product instructions. Then the NanoDrop spectrophotometer was used to determine the concentration and quality of the extracted DNA. Genomic DNA was re-extracted for samples that did not meet the quality requirements. The 16S ribosomal DNA (rDNA) V4 region was amplified by using the following primers: 515F, 5’-GTG CCA GCMGCC GCG GTAA-3’; 806R, 5’-GGA CTA CVSGGG TAT CTAAT-3’. The IlluminaHiSeq2000 platform was used for sequencing. The sequencing results have been deposited in GenBank (accession number PRJNA768326).

### High-Throughput Sequencing

The data were subjected to sequence denoising or operational taxonomic unit (OTU) clustering by using the analysis process of the Vsearch software or QIIME2 DADA2 analysis. The Vsearch method (20) mainly includes de-priming, splicing, quality filtering, de-duplication, de-chimerism, clustering and other steps to obtain OTU. The DADA2 method mainly carries out the steps of depriming, quality filtering, de-noising, splicing and de-chimerism. Each deduplicated sequence generated after DADA2 quality control is called an amplicon sequence variant (ASV), or feature sequence (corresponding to a representative sequences of OTU). ASV and OTU were then used to analyse the species composition, alpha diversity, beta diversity, species difference analysis and symbol species, etc.

### Statistical Analysis

GraphPad Prism 7 was used for statistical analysis. The data were expressed as the mean ± standard deviation (SD). One-way analysis of variance (ANOVA) was used to determine a statistically significant difference (p < 0.05)

## Results

### Probiotic Properties

**Figure 1:**
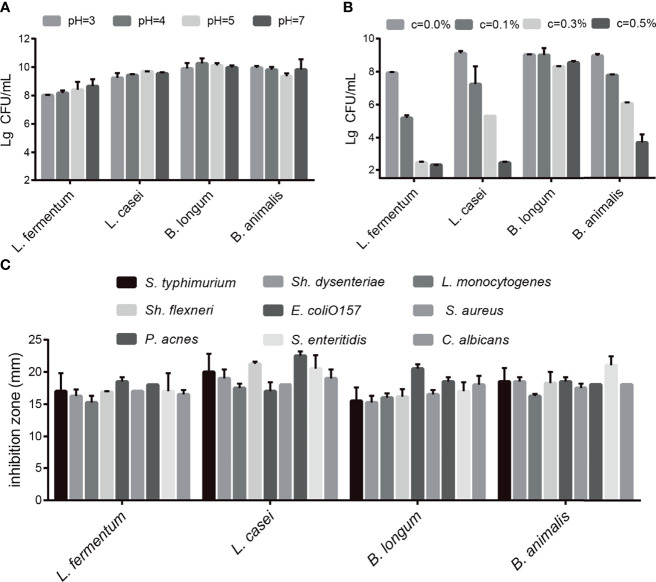
Evaluation of the probiotic characteristics of *L. fermentum* SX-0718, *L. casei* SX-1107, *Bifidobacterium* SX-1326, and *B animalis* SX-0582. **(A)** The acid tolerance of *L. fermentum* SX-0718, *L. casei* SX-1107, *B longum* SX-1326 and *B animalis* SX-0582. **(B)** The cholate tolerance of *L. fermentum* SX-0718, *L. casei* SX-1107, *B longum* SX-1326 and *B animalis* SX-0582. **(C)** Antibacterial activities of *L. fermentum* SX-0718, *L. casei* SX-1107, *B longum* SX-1326 and *B animalis* SX-0582 on *Salmonella typhimurium* ATCC 13311, *Shigella flexneri* ATCC 12022, *P ropionibacterium acnes* ATCC 11827, *Sh. dysenteriae* 301, *Escherichia coli* O157, *S. enteritidis* ATCC 13076, *Listeria monocytogenes* ATCC 19111, *Staphylococcus aureus* Cowan1 and *Candida albicans* SC531.

First, we conducted acid tolerance, bile salt tolerance and antibacterial experiments to evaluate the probiotic characteristics of the selected strains. The four selected probiotics were tolerant to medium strong acid (**Figure 1A**) and survived in different bile salt concentrations (**Figure 1B**). As shown in **Figure 1A**, after 4 h in pH 3 PBS, the survival rates of *L. fermentum* SX-0718, *L. casei* SX-1107, *B. longum* SX-1326, and *B. animalis* SX-0582 were 80.2%, 92.4%, 99.0%, and 99.5%, respectively. As shown in **Figure 1B**, *L. fermentum SX-0718* had low tolerance to bile salt. *B. longum* SX-1326 showed excellent bile salt tolerance: at a concentration of 0.5%, the survival rate was still 85.4%. In addition, the selected probiotics significantly inhibited the growth of the pathogens *S. typhimurium* ATCC 13311, *Sh. flexneri* ATCC 12022, *P. acnes* ATCC 11827, *Sh. dysenteriae* 301, *E. coli* O157, *S. enteritidis* ATCC 13076, *L. monocytogenes* ATCC 19111, *Staph. aureus* Cowan1 and *C. albicans* SC531, with a suppression area from 15.25 mm to 22.5 mm (**Figure 1C**).

### The Probiotic Combination Improved Behaviour in SAMP8 Mice

**Figure 2:**
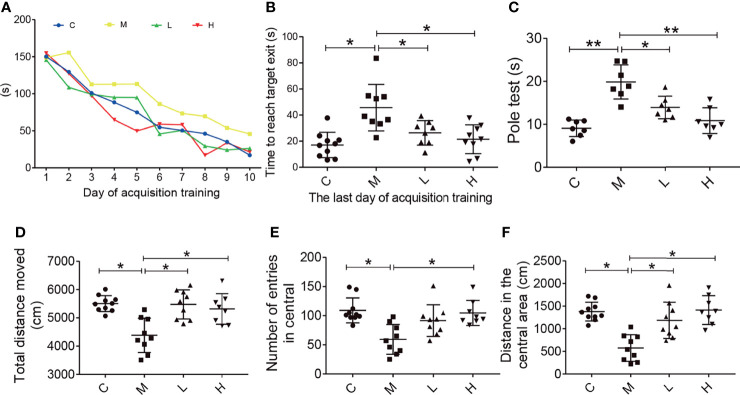
The probiotic combination improved spatial memory, motor dysfunction, and exploratory behaviour in SAMP8 mice. **(A)** The time to reach the target exit during the training test (the Barnes maze). **(B)** The probiotic combination reduced time it took SAMP8 mice to reach the target exit during the probe test of the Barnes maze. **(C)** The probiotic combination improved the motor dysfunction in SAMP8 mice (the pole test). **(D)** The probiotic combination increased the total distance in SAMP8 mice moved in the open-field test. **(E)** The probiotic combination increased the number of entries SAMP8 mice made in central area of the open-field test. **(F)** The probiotic combination increased the distance SAMP8 mice moved in the central area of the open-field test. C: control group (n = 10), M: model group (n = 9), L: low-dose probiotic group (n = 8), H: high-dose probiotic group (n = 9). Data are presented as the means ± SD. *p < 0.05 **p < 0.01.

The Barnes maze test was used to evaluate the effect of the probiotic combination on the spatial learning and memory abilities. Compared with C group, in daily training, mice in group M took significantly longer time to find the platform, while probiotic combination (group L and H) greatly reduced the escape latency, the latency period of searching for right foramen. On the probe test, the latency of the probiotic combination treatment was significantly shorter than that of the M group (L vs. M = 27.4 s vs. 48.19 s; p < 0.001; H vs. M = 20.34 s vs. 48.19 s; p < 0.001), and there was no significant difference between low-dose and high-dose probiotics (**Figure 2A**).

The pole test was conducted to observe the effect of the probiotic combination on the motor dysfunction of SAMP8 mice. As shown in **Figure 2**, compared with the C group, the M group showed significant motor retardation (M vs. C = 19.46 s vs. 8.877 s; p < 0.001), and treatment with either low or high concentrations of the probiotic combination significantly alleviated the motor dysfunction of SAMP8 mice (L vs. M = 13.78 s vs. 19.46 s; p < 0.001; H vs. M = 10.73 s vs. 19.46 s; p < 0.001).

The open field test was used to detect the behavioral and mental changes of mice to the new environment, such as exploratory behavior and anxiety. Experimental parameters showed that compared with mice in group M (distance = 4384 cm, number = 109, distance = 574.6), mice in groups C, L, and H had a longer total movement distance (distance = 5505 cm, distance = 5474 cm, distance = 5313 cm; C vs M, P < 0.05; L vs M, p < 0.05; H vs M, p < 0.05), more times to enter the central area (number = 59.33, number = 91.56, number = 104.8; C vs M, p < 0.05; L vs M, p < 0.05; H vs M, p < 0.05) and longer central movement distance (distance = 1379 cm, distance = 1187 cm, distance = 1416 cm; C vs M, p < 0.05; L vs M, p < 0.05; H vs M, p < 0.05). In addition, there was no dose-dependent in probiotics treatment.

### The Probiotic Combination Reduced Neuronal Death and Prevented the Decrease of Sirt 1 Expression in the Hippocampus of SAMP8 Mice

**Figure 3:**
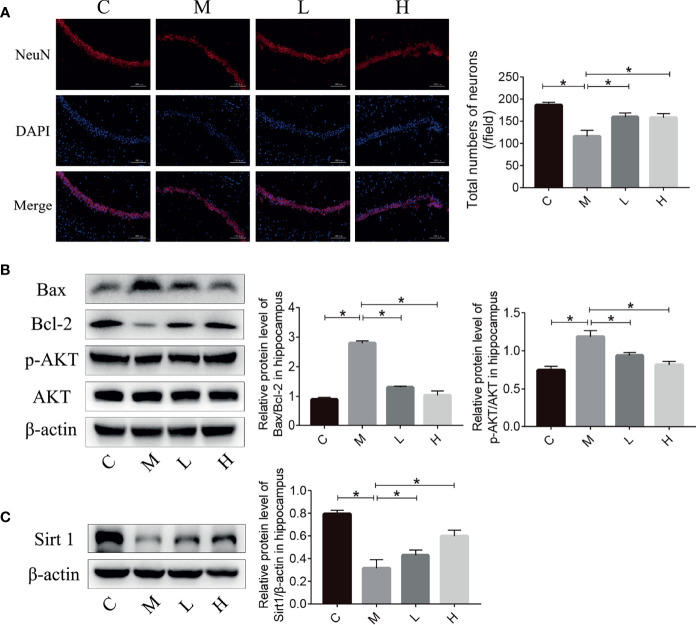
The probiotic combination reduced neuronal death and prevented the decrease in Sirt 1 expression in the hippocampus of SAMP8 mice. **(A)** Photomicrographs and quantitative analysis of the number of neurons in the hippocampus, analysed by NeuN immunostaining. **(B)** The effect of the probiotic combination on the expression levels of Bax, Bcl-2, p-AKT and AKT in the hippocampus. **(C)** The effect of the probiotic combination on the expression level of Sirt 1 in the hippocampus. C: control group (n = 4), M: model group (n = 4), L: low-dose probiotics group (n = 4), H: high-dose probiotics group (n = 4). Data are presented as the means ± SD. *p < 0.05.

The reduction in the number of hippocampal neurons in SAMP8 mice is closely related to the learning and memory declines. Therefore, we detected the number of neurons in the hippocampus of SAMP8 mice through NeuN immunostaining. Compared with C group, the M group had fewer hippocampal neurons; the probiotic combination protected hippocampal neurons compared with the M group (**Figure 3A**). Neuronal loss due to apoptosis – regulated by Bax and Bcl-2 – plays an important part in the occurrence and development of aging. The p-AKT can modulate apoptosis by regulating Bcl-2/Bax. We evaluated the expression of Bax, Bcl-2, and p-AKT in the hippocampus of mice by using western blot. The results showed that the expression of pro-apoptotic protein Bax in group M mice was significantly higher than that in C mice, and the expression of anti-apoptotic protein Bcl-2 and p-AKT was significantly lower than that of the M group, while the pro-apoptotic protein expression in the L and H groups was significantly lower than that in M group, and the expression of apoptotic protein was notably higher than that of the M group. In addition, the expression of Sirt 1 in the C, L and H groups was prominently higher than that in the M group, indicated that probiotic combination could inhibit the decrease of Sirt 1 expression in aging mice.

### The Probiotic Combination Inhibited TLR4/NF-κB Signaling Pathway and Hippocampal Inflammation in SAMP8 Mice

**Figure 4:**
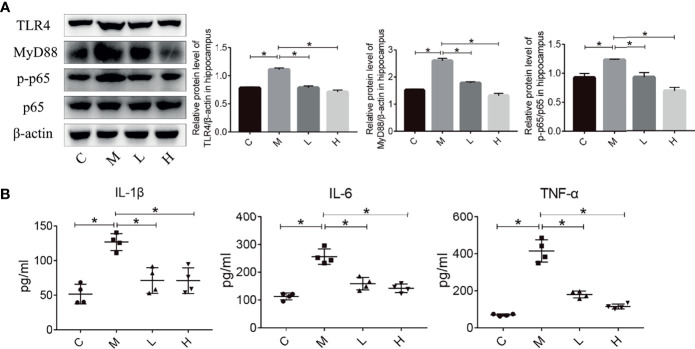
The probiotic combination modulated the TLR4/NFkB signalling pathway and hippocampal inflammation in SAMP8 mice. **(A)** The effect of the probiotic combination on the expression levels of TLR4, MyD88, p-p65 and p65 in the hippocampus. **(B)** The probiotic combination reduced the levels of the pro-inflammatory cytokines IL-1β, IL-6 and TNF-α. C: control group (n = 4), M: model group (n = 4), L: low-dose probiotics group (n = 4), H: high-dose probiotics group (n = 4). Data are presented as the means ± SD. *p < 0.05.

Inflammation plays an important role in maintaining and promoting aging, and the TLR4/NFκB pathway is closely related to inflammation. Hence, we evaluated the expression of proteins involved in the TLR4/NFκB signalling pathway in the hippocampus by using western blot. Compared with the M group, the probiotic combination significantly reduced TLR4, MyD88 and p-p65/p65 expression (**Figure 4A**, p < 0.01). In addition, the detection of inflammatory factor protein levels showed that probiotic combination significantly reduced the relative expression of the pro-inflammatory cytokine IL-1β, IL-6 and TNF-α compared with the M group, (**Figure 4B**; p<0.01). These results suggest that the probiotic combination modulates the upregulation of TLR4/NFκB signalling pathway components to reduce inflammation in SAMP8 mice.

### The Probiotic Combination Altered the Gut Microbiota Composition of SAMP8 Mice

**Figure 5:**
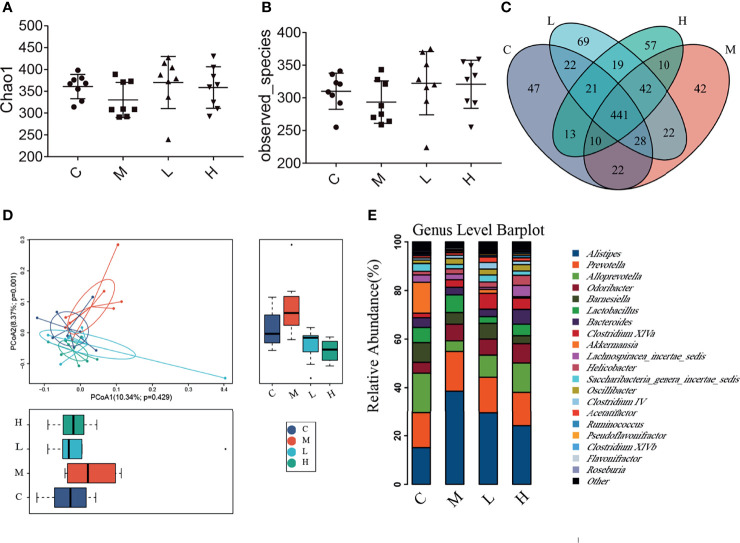
The probiotic combination altered the gut microbiota composition of SAMP8 mice. **(A)** The Chao1 index. **(B)** The Observed_species index. **(C)** The Venn diagram. **(D)** The PCoA analysis. **(E)** Species abundance analysis at the genus level. C, control group (n = 8); M, model group (n = 8); L, low-dose probiotics group (n = 8); H, high-dose probiotics group (n = 8). Data are presented as the means ± SD.

The intestinal microbiota is closely related to the occurrence and development of senescence. We collected the faeces of mice and analysed their intestinal microbiota changes by using high-throughput sequencing. A total of 1,138,968 valid tags and 10,202 OTUs were obtained, with an average of 2550.25 per group (data not shown). To analyse the influence of the probiotic combination on the intestinal microbiota of SAMP8 mice, we carried out a diversity analysis of the microbiota. The Observed_species index represented the actual observed number of OTU, and the Chao1 index indicates the diversity of the microbiota. As shown in the **Figure 5**, the Observed_species index and Chao1 indexes of the M group of mice were reduced, while the probiotic combination increased the abundance and diversity of intestinal microbiota, although the changes were not significant (**Figures 5A** **, B**). The Venn diagram shows 441 common OTU among all the groups, with 47, 42, 69, and 57 unique OUT for the C, M, L, and H groups, respectively (**Figure 5C**). In addition, the principal co-ordinates analysis (PCoA) used to study the similarity of microbial communities showed that the points of the C group are clustered together, the points of the M group are relatively scattered, and the samples of the C, L, and H groups have high similarity. These findings indicate that the probiotic combination alters the intestinal microbiota of aging mice to a pattern like that of normal mice (**Figure 5D**). We analysed the relative abundance of the top 20 bacterial genera among the different groups. There was notably increased the relative abundance of *Alisipes*, *Prevotella*, *Odoribacter*, *Lactobacillus* and *Oscillibacter*, and decreased the relative abundance of *Alloprevotella*, *Barnesiella* and *Akkermansia*. Compared with the M group, the probiotic combination reduced the relative abundance of *Alistipes* and *Prevotella*, and increased the relative abundance of *Alloprevotella*, *Acetatifactor*, and *Clostridium* XIVa (**Figure 5E**).

### The Probiotic Combination Alleviated Colonic Inflammation and Decreased Tight Junction Protein Expression in SAMP8 Mice

**Figure 6:**
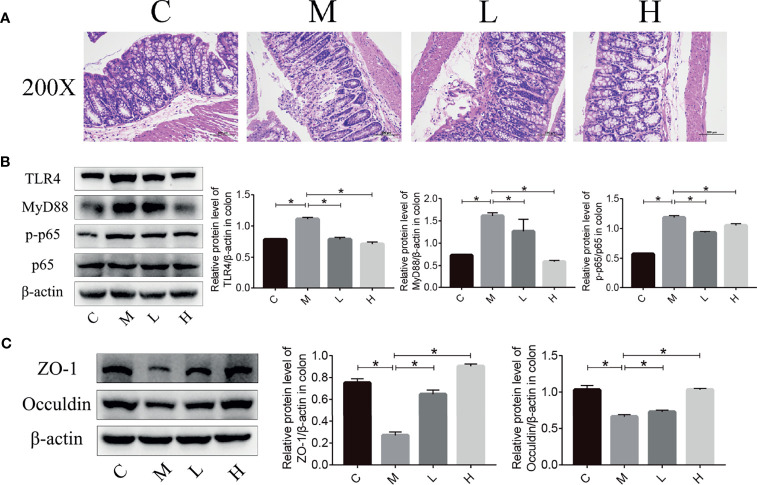
The probiotic combination alleviated colonic inflammation and decreased tight junction protein expression in SAMP8 mice. **(A)** HE staining of the colon. **(B)** The effect of the probiotic combination on the expression levels of TLR4, MyD88, p-p65 and p65 in the colon. **(C)** The effect of the probiotic combination on the expression levels of ZO-1 and Occuldin in the colon. C, control group (n = 4); M, model group (n = 4); L, low-dose probiotics group (n = 4); H, high-dose probiotics group (n = 4). Data are presented as the means ± SD. *p < 0.05.

Intestinal microbiota imbalance and intestinal inflammation can be causally related to each other. Therefore, haematoxylin and eosin (H&E) staining was used to detect the pathological changes in the mice colon, and the western blot were applied to detect the expression of key proteins in the TLR4/NFκB inflammatory pathway and intestinal permeability-related proteins in mouse colon tissue. As shown in **Figure 6**, mucosal epithelial cells were shed and a small amount of the intestinal gland structure was destroyed in the colon of mice in the M group, while no mucosal epithelial cells were shed and the intestinal gland structure was maintained in the intestinal tissues of the C, L and H groups. In addition, we used western blot to examine the expression of key proteins in the TLR4/NFκB inflammatory pathway as well as proteins related to intestinal permeability. Compared with the C group, ZO-1 and Occuldin expression decreased and TLR4, MyD88, and p-p65 expression increased in the M group. The probiotic combination significantly increased the expression of ZO-1 and Occuldin – indicating increased integrity of the intestinal barrier – and reduced the expression of TLR4, MyD88, and p-p65 – indicating a modulation of intestinal inflammation (**Figures 6B** **, C**).

## Discussion

The aging of the population is a prominent global problem in today’s society. Aging can lead to a series of physical changes and abnormal behaviours, such as hair loss, sagging skin, reduced mental ability, decreased memory and even dementia (21). The health problems caused by the aging are major challenges in the field of health care (12). There is currently no good way to prevent or treat aging. Therefore, research related to anti-aging treatments and aging-related diseases is needed urgently. The SAMP8 mouse exhibits rapid aging that is consistent with what happens in humans. It is mainly characterised by reduced learning and memory, cognitive impairment and neurodegenerative changes. It is a common natural pathogenesis model to study dementia and aging (22).

In this research, the SAMP8 mice were used to evaluate the effects of a probiotic combination. First, we identified four probiotics (*L. fermentum* SX-0718*, L. casei* SX-1107*, B. longum* SX-1326, and *B. animalis* SX-0582; Jiangxi Shanxing Biotechnology Co., Ltd, Nanchang, Jiangxi, PR China) from the faeces of centenarians. *In vitro* experiments verified that they had good acid resistance, bile salt resistance and antibacterial properties (**Figure 1**). In addition, the probiotic combination could alleviate weight loss and reduce the aging score of SAMP8 mice (**Supplementary Figures 1B, C**). In behavioural tests, aging mice showed impaired spatial memory, motor dysfunction and decreased exploratory behaviour (**Figure 2**). The probiotic combination was able to ameliorate these changes, indicating that it may exert anti-aging effects.

Given the promising behavioural data, we studied the possible anti-aging mechanism of the probiotic combination. The hippocampus is involved in learning and memory and damage to this brain region can cause learning and memory impairment and spatial positioning disorders (23). Compared with the C group, there were fewer neurons in the hippocampus of the M group. The probiotic combination could alleviate the loss of neurons in SAMP8 mice (**Figure 3A**). Many studies have shown that the AKT signalling pathway plays a significant role in the apoptosis and metabolism of neurons. The AKT signalling pathway improves cell survival by regulating forkhead transcription factor (FKHR), as well as mammalian target of rapamycin (mTOR) to increase protein synthesis and affect nerves. The growth of synapses and the development of synaptic plasticity ultimately affect learning and memory functions (24, 25). The p-AKT can directly regulate formation of the Bcl-2/Bax heterodimer through phosphoinositide 3-kinase (PI3K)-1 (26). Sirt 1 plays a significant role in the development of the brain and neurons as well as the pathogenesis of AD. Neurodegeneration and cognitive function are significantly improved after injection of a virus encoding Sirt 1 into the hippocampus (27). Research by Zhou et al. (28) also showed that blocking Sirt 1 with small interfering RNA (siRNA) can accelerate AD pathology and cognitive impairment. Moreover, there is an interaction between Sirt 1 and AKT. Histone acetyltransferase (HAT) can inhibit AKT phosphorylation, while Sirt 1 is a histone deacetylase (HDAC) that uses NAD to deacetylate AKT on lysine residues targeted by HAT (29). Sirt 6 is another NAD+ dependent deacetylase, Kawahara et al. (30) found that Sirt 6 can be recruited to the promoters of NFκB downstream genes, reducing the level of promoter acetylation and inhibiting the expression of downstream genes related to apoptosis and cellular senescence. Studies have shown that overexpression of Sirt 6 can increase the healthy lifespan of mice by an average of 30% (31), so Sirt 6 may be another mechanism by which probiotics exert anti-aging effects, although this eventuality requires further study. The detection of NeuN-positive cells, apoptosis, proliferation-related proteins, and Sirt 1 in the hippocampus demonstrated that the probiotic combination can prevent hippocampal neuronal loss (**Figure 3**). The neuroprotective effect of the probiotic combination on hippocampal neurons may be through the regulation of the AKT signalling pathway and Sirt 1.

Neuroinflammation plays an important part in aging-related neurological diseases such as AD and PD (32). In these diseases, various inflammatory factors (food antigens, lipopolysaccharides, free fatty acids, reactive oxygen species, etc.) bind to TLR and activate NFκB, an important factor in the immune system and the inflammatory process, after signal transduction. This phenomenon leads to the release of many pro-inflammatory factors (IL-1β, IL-6, TNF-α, etc.), causing neuroinflammation, damage to the brain, and neuronal death (32, 33). Some drugs can alleviate the progression of neurodegenerative diseases such as AD and PD by modulating neuroinflammation (34, 35). In this study, compared with the M group, the levels of TLR4, MyD88, p-p65/p65, IL-1β, TNF-α and IL-6 were significantly reduced in the L and H groups (**Figure 4**), indicating that the probiotic combination can inhibit neuroinflammation in aging mice and protect neurons.

The intestinal microbiota is closely related to human health. Under normal physiological conditions, the host and the intestinal microbiota maintain an ecological balance through synergistic antagonism. Local and continuous abnormal activities of intestinal microbes can cause inflammation of the intestinal mucosa. However, local inflammatory reactions often lead to the occurrence of chronic low-grade inflammation throughout the body. Chronic low-grade inflammation in the intestinal mucosa will result in the destruction of the intestinal barrier, and many harmful factors (lipopolysaccharides, pathogenic bacteria, etc.) will enter the body to induce neuroinflammation (14, 16, 32). We detected changes in the intestinal microbes of mice by using high-throughput sequencing. Compared with the C group, the diversity and richness of the intestinal microbiota in the M group was decreased. The probiotic combination counteracted these changes in the intestinal microbiota and promoted the restoration of intestinal homeostasis (**Figures 5A** **, B**). Based on PCoA, we found that after treatment with the probiotic combination, the intestinal microbiota of the aging mice aggregated with the intestinal microbiota of the C group (**Figure 5D**), which indicates that the probiotic combination restores the intestinal microbiota of the aging mice to the composition of normal mice. Consistently with the findings of others, the abundance of *Akkermansia muciniphila* was decreased and the abundance of *Prevotella* was increased in aging mice (15). Moreover, the probiotic combination reduced the relative abundance of *Alistipes* and *Prevotella* (**Figure 5E**). *Akkermansia* can improve metabolism, exerts anti-inflammatory activity, and augments the efficacy of immunotherapy; it is also closely related to the intestinal barrier function. The decline in *Akkermansia* may cause damage to the intestinal barrier and lead to inflammation in the body (36). *Alistipes* is a gram-negative anaerobe and a facultative pathogen. The abundance of *Alistipes* increases in patients with depression (37). In the gut microbes of patients with intestinal irritability syndrome, *Alistipes* tends to increase, and this change may be one of the underlying causes of depression in such patients (38). Researchers have found that *Alistipes* can affect the use of tryptophan, impairing the balance of the serotonergic system in the intestine, and then inhibiting brain electrical activity, which may lead to cognitive dysfunction (37). *Prevotella* is a genus of essential bacteria in a healthy intestinal biota. However, studies have shown that *Prevotellaceae* bacteria increase in the intestinal microbiota of patients with schizophrenia (39). The abundance of *Prevotella* in the intestinal microbiota of patients with cerebral palsy and children with cerebral palsy and epilepsy increases significantly (40). The increased abundance of *Prevotella* may be related to diseases such as periodontitis, bacterial vaginosis, rheumatoid arthritis, metabolic disorders and low-grade systemic inflammation (41–43). This may be due to the intestinal colonisation of *Prevotella* leading to changes in the metabolism of the microbiota, reducing the production of IL-18, which intensifies intestinal inflammation and may lead to systemic autoimmunity (44).

Studies have shown that intestinal barrier dysfunction, and thus chronic inflammation from the intestine, plays an important role in aging (45). Therefore, we examined the pathological changes of colon in mice and detected the expression of components of the TLR4/NFκB inflammatory pathway (TLR4, MyD88 and p-p65) and tight junction proteins (ZO-1 and Occuldin) in the colon. The mucosal epithelial cells in the colon of aging mice were shed and a small amount of intestinal gland structure was destroyed. The probiotic combination counteracted these changes (**Figure 6A**). Compared with the C group, the TLR4/NFκB signalling pathway was activated in the M group, with increased the expression of the inflammation-related proteins TLR4 and MyD88, which in turn increased the level of p-p65 (**Figure 6B**). At the same time, the expression of ZO-1 and Occuldin decreased significantly (**Figure 6C**). Treatment with the probiotic combination relieved the intestinal inflammation and increased the integrity of the intestinal barrier.

**Figure 7:**
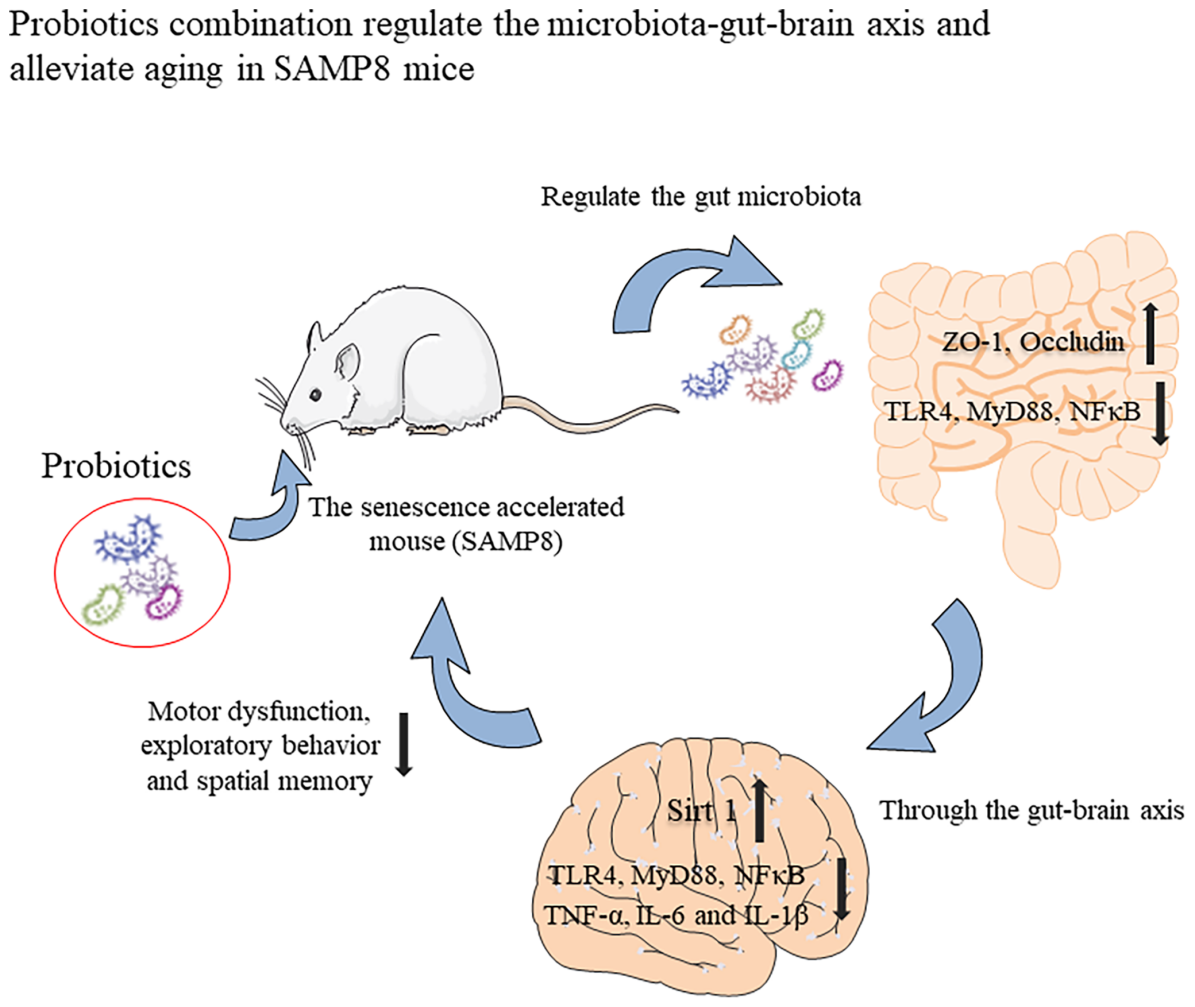
Graphical summary of this study. The probiotic combination, determined by screening the faeces of centenarians, can increase the expression of intestinal permeability-related proteins ZO-1 and Occuldin by regulating the intestinal microbiota. It also inhibits TLR4/NFκB-induced intestinal inflammation and then inhibits TLR4/NFκB-induced neuroinflammation through the gut-brain axis and up-regulates the expression of Sirt 1 to protect hippocampal neurons. These changes underlie the improved spatial memory, motor function, and exploratory behaviour of aging mice.

In summary, we have shown that the probiotic combination, developed by screening the faeces of centenarians, increases the expression of intestinal permeability-related proteins ZO-1 and Occuldin by regulating the intestinal microbiota. It also inhibits TLR4/NFκB-induced intestinal inflammation and consequently, neuroinflammation through the gut-brain axis and upregulates the expression of Sirt 1 to protect hippocampal neurons (**Figure 7**). These changes underlie improved spatial memory, motor function and exploratory behaviour of aging mice. Our research provides the basis for the probiotic combination to become a dietary supplement for anti-aging.

## Data Availability Statement

The data presented in the study are deposited in the GenBank repository, accession number PRJNA768326.

## Ethics Statement

The animal study was reviewed and approved by the Laboratory Animal Ethics Committee of Nanchang Royo Biotech Co., Ltd (license No. RYE2020051401) on May 14, 2020.

## Author Contributions

XF and TC contributed to conception and design of the study. XZ, MY, and JW performed the experiments. YW, DH, and BW carried out data analysis. All authors participated in drafting of the manuscript and critical revision of the draft and contributed to the article and approved the submitted version.

## Funding

This work was supported by grants from the National Natural Science Foundation of China (Nos. 82060638, 82060222), Science and Technology Plan of Jiangxi Health Planning Committee (Nos. 20195092), the Science and Technology Project of Jiangxi (Nos. 20194BCJ22032, 20192BBG70031), Nanchang Hongcheng Project to TC, and dou-ble 10-thousand plan of Jiangxi Province (innovation and technology professionals as the high-end talent).

## Conflict of Interest

The authors declare that the research was conducted in the absence of any commercial or financial relationships that could be construed as a potential conflict of interest.

## Publisher’s Note

All claims expressed in this article are solely those of the authors and do not necessarily represent those of their affiliated organizations, or those of the publisher, the editors and the reviewers. Any product that may be evaluated in this article, or claim that may be made by its manufacturer, is not guaranteed or endorsed by the publisher.

## Supplementary Material

Supplementary Figure 1 The probiotic combination delayed the decrease in body weight and the increase in the aging score of SAMP8 mice. **(A)** The experimental design of this study. **(B)** Weekly body weight changes of SAMP8 mice during gavage of the probiotic combination. **(C)** The aging degree scores of SAMP8 mice after gavage of the probiotic combination. C: control group (n = 10), M: model group (n = 9), L: how-dose probiotics group (n = 8), H: high-dose probiotics group (n = 9). Data are presented as the means ± SD. *p < 0.05 **p < 0.0.

## References

[1] Cano M Guerrero-Castilla A Nabavi SM Ayala A Argüelles S. Targeting Pro-Senescence Mitogen Activated Protein Kinase (Mapk) Enzymes With Bioactive Natural Compounds. Food Chem Toxicol an Int J Published Br Ind Biol Res Assoc (2019) 131:110544. doi: 10.1016/j.fct.2019.05.052 31201898

[2] Morelli N Barello S Mayan M Graffigna G. Supporting Family Caregiver Engagement in the Care of Old Persons Living in Hard to Reach Communities: A Scoping Review. Health Soc Care Commun (2019) 27:1363–74. doi: 10.1111/hsc.12826 31441179

[3] McHugh D Gil J. Senescence and Aging: Causes, Consequences, and Therapeutic Avenues. J Cell Biol (2018) 217:65–77. doi: 10.1083/jcb.201708092 29114066 PMC5748990

[4] Mattson MP Arumugam TV. Hallmarks of Brain Aging: Adaptive and Pathological Modification by Metabolic States. Cell Metab (2018) 27:1176–99. doi: 10.1016/j.cmet.2018.05.011 PMC6039826 29874566

[5] Nie X Chen Y Li W Lu Y. Anti-Aging Properties of Dendrobium Nobile Lindl.: From Molecular Mechanisms to Potential Treatments. J Ethnopharmacol (2020) 257:112839. doi: 10.1016/j.jep.2020.112839 32268205

[6] Guedj A Volman Y Geiger-Maor A Bolik J Schumacher N Künzel S. Gut Microbiota Shape 'Inflamm-Ageing' Cytokines and Account for Age-Dependent Decline in DNA Damage Repair. Gut (2020) 69:1064–75. doi: 10.1136/gutjnl-2019-318491 31586932

[7] Michaud M Balardy L Moulis G Gaudin C Peyrot C Vellas B. Proinflammatory Cytokines, Aging, and Age-Related Diseases. J Am Med Directors Assoc (2013) 14:877–82. doi: 10.1016/j.jamda.2013.05.009 23792036

[8] Morshedi M Hashemi R Moazzen S Sahebkar A Hosseinifard ES. Immunomodulatory and Anti-Inflammatory Effects of Probiotics in Multiple Sclerosis: A Systematic Review. J Neuroinflamm (2019) 16:231. doi: 10.1186/s12974-019-1611-4 PMC6868771 31752913

[9] López-Otín C Blasco MA Partridge L Serrano M Kroemer G. The Hallmarks of Aging. Cell (2013) 153:1194–217. doi: 10.1016/j.cell.2013.05.039 PMC3836174 23746838

[10] Kalogeropoulos A Georgiopoulou V Psaty BM Rodondi N Smith AL Harrison DG. Inflammatory Markers and Incident Heart Failure Risk in Older Adults: The Health ABC (Health, Aging, and Body Composition) Study. J Am Coll Cardiol (2010) 55:2129–37. doi: 10.1016/j.jacc.2009.12.045 PMC3267799 20447537

[11] Bruunsgaard H Ladelund S Pedersen AN Schroll M Jørgensen T Pedersen BK. Predicting Death From Tumour Necrosis Factor-Alpha and Interleukin-6 in 80-Year-Old People. Clin Exp Immunol (2003) 132:24–31. doi: 10.1046/j.1365-2249.2003.02137.x 12653832 PMC1808682

[12] Haran JP McCormick BA. Aging, Frailty, and the Microbiome-How Dysbiosis Influences Human Aging and Disease. Gastroenterol (2021) 160:507–23. doi: 10.1053/j.gastro.2020.09.060 PMC7856216 33307030

[13] Quigley EMM. Microbiota-Brain-Gut Axis and Neurodegenerative Diseases. Curr Neurol Neurosci Rep (2017) 17:94. doi: 10.1007/s11910-017-0802-6 29039142

[14] Wang HX Wang YP. Gut Microbiota-Brain Axis. Chin Med J (2016) 129:2373–80. doi: 10.4103/0366-6999.190667 PMC5040025 27647198

[15] Bárcena C Valdés-Mas R Mayoral P Garabaya C Durand S Rodríguez F. Healthspan and Lifespan Extension by Fecal Microbiota Transplantation Into Progeroid Mice. Nat Med (2019) 25:1234–42. doi: 10.1038/s41591-019-0504-5 31332389

[16] Białecka-Dębek A Granda D Szmidt MK Zielińska D. Gut Microbiota, Probiotic Interventions, and Cognitive Function in the Elderly: A Review of Current Knowledge. Nutrients (2021) 13:2514. doi: 10.3390/nu13082514 34444674 PMC8401879

[17] Deng K Chen T Wu Q Xin H Wei Q Hu P. *In Vitro* and *In Vivo* Examination of Anticolonization of Pathogens by Lactobacillus Paracasei FJ861111.1. J Dairy Sci (2015) 98:6759–66. doi: 10.3168/jds.2015-9761 26254535

[18] Xia C Cao X Cui L Liu H Wang S Chen T. Anti-Aging Effect of the Combination of Bifidobacterium Longum and B. Animalis in a D-Galactose-Treated Mice. J Funct Foods (2020) 69:103938. doi: 10.1016/j.jff.2020.103938

[19] Hosokawa M Kasai R Higuchi K Takeshita S Shimizu K Hamamoto H. Grading Score System: A Method for Evaluation of the Degree of Senescence in Senescence Accelerated Mouse (SAM). Mech Ageing Dev (1984) 26:91–102. doi: 10.1016/0047-6374(84)90168-4 6748759

[20] Rognes T Flouri T Nichols B Quince C Mahé F. VSEARCH: A Versatile Open Source Tool for Metagenomics. PeerJ (2016) 4:e2584. doi: 10.7717/peerj.2584 27781170 PMC5075697

[21] da Costa JP Vitorino R Silva GM Vogel C Duarte AC. Rocha-Santos T. A Synopsis on Aging-Theories, Mechanisms and Future Prospects. Ageing Res Rev (2016) 29:90–112. doi: 10.1016/j.arr.2016.06.005 27353257 PMC5991498

[22] Liu B Liu J Shi JS. SAMP8 Mice as a Model of Age-Related Cognition Decline With Underlying Mechanisms in Alzheimer's Disease. J Alzheimer's Dis JAD (2020) 75:385–95. doi: 10.3233/jad-200063 32310176

[23] Lazarov O Hollands C. Hippocampal Neurogenesis: Learning to Remember. Prog Neurobiol (2016) 138-140:1–18. doi: 10.1016/j.pneurobio.2015.12.006 26855369 PMC4828289

[24] Jo H Mondal S Tan D Nagata E Takizawa S Sharma AK. Small Molecule-Induced Cytosolic Activation of Protein Kinase Akt Rescues Ischemia-Elicited Neuronal Death. Proc Natl Acad Sci USA (2012) 109:10581–6. doi: 10.1073/pnas.1202810109 PMC3387065 22689977

[25] Fournier NM Lee B Banasr M Elsayed M Duman RS. Vascular Endothelial Growth Factor Regulates Adult Hippocampal Cell Proliferation Through MEK/ERK- and PI3K/Akt-Dependent Signaling. Neuropharmacol (2012) 63:642–52. doi: 10.1016/j.neuropharm.2012.04.033 PMC3392414 22580375

[26] Li H Tang Z Chu P Song Y Yang Y Sun B. Neuroprotective Effect of Phosphocreatine on Oxidative Stress and Mitochondrial Dysfunction Induced Apoptosis *In Vitro* and *In Vivo*: Involvement of Dual PI3K/Akt and Nrf2/HO-1 Pathways. Free Radical Biol Med (2018) 120:228–38. doi: 10.1016/j.freeradbiomed.2018.03.014 29559323

[27] Kim D Nguyen MD Dobbin MM Fischer A Sananbenesi F Rodgers JT. SIRT1 Deacetylase Protects Against Neurodegeneration in Models for Alzheimer's Disease and Amyotrophic Lateral Sclerosis. EMBO J (2007) 26:3169–79. doi: 10.1038/sj.emboj.7601758 PMC1914106 17581637

[28] Zhou Y Zhu F Liu Y Zheng M Wang Y Zhang D. Blood-Brain Barrier-Penetrating siRNA Nanomedicine for Alzheimer's Disease Therapy. Sci Advances (2020) 6:7031. doi: 10.1126/sciadv.abc7031 PMC7546706 33036977

[29] Lin JY Kuo WW Baskaran R Kuo CH Chen YA Chen WS. Swimming Exercise Stimulates IGF1/ PI3K/Akt and AMPK/Sirt1/Pgc1α Survival Signaling to Suppress Apoptosis and Inflammation in Aging Hippocampus. Aging (2020) 12:6852–64. doi: 10.18632/aging.103046 PMC7202519 32320382

[30] Kawahara TL Michishita E Adler AS Damian M Berber E Lin M. SIRT6 Links Histone H3 Lysine 9 Deacetylation to NF-kappaB-Dependent Gene Expression and Organismal Life Span. Cell (2009) 136:62–74. doi: 10.1016/j.cell.2008.10.052 19135889 PMC2757125

[31] Roichman A Elhanati S Aon MA Abramovich I Di Francesco A Shahar Y. Restoration of Energy Homeostasis by SIRT6 Extends Healthy Lifespan. Nat Commun (2021) 12:3208. doi: 10.1038/s41467-021-23545-7 34050173 PMC8163764

[32] Sampson TR Debelius JW Thron T Janssen S Shastri GG Ilhan ZE. Gut Microbiota Regulate Motor Deficits and Neuroinflammation in a Model of Parkinson's Disease. Cell (2016) 167:1469–80.e12. doi: 10.1016/j.cell.2016.11.018 27912057 PMC5718049

[33] Megur A Baltriukienė D Bukelskienė V Burokas A. The Microbiota-Gut-Brain Axis and Alzheimer's Disease: Neuroinflammation Is to Blame? Nutrients (2020) 13:37. doi: 10.3390/nu13010037 PMC7824474 33374235

[34] Yang Q Luo L Sun T Yang L Cheng LF Wang Y. Chronic Minocycline Treatment Exerts Antidepressant Effect, Inhibits Neuroinflammation, and Modulates Gut Microbiota in Mice. Psychopharmacol (2020) 237:3201–13. doi: 10.1007/s00213-020-05604-x 32671421

[35] Dong Y Li X Cheng J Hou L. Drug Development for Alzheimer's Disease: Microglia Induced Neuroinflammation as a Target? Int J Mol Sci (2019) 20:558. doi: 10.3390/ijms20030558 PMC6386861 30696107

[36] Geerlings SY Kostopoulos I de Vos WM Belzer C. Akkermansia Muciniphila in the Human Gastrointestinal Tract: When, Where, and How? Microorganisms (2018) 6:75. doi: 10.3390/microorganisms6030075 PMC6163243 30041463

[37] Ait-Belgnaoui A Colom A Braniste V Ramalho L Marrot A Cartier C. Probiotic Gut Effect Prevents the Chronic Psychological Stress-Induced Brain Activity Abnormality in Mice. Neurogastroenterol Motil Off J Eur Gastrointestinal Motil Soc (2014) 26:510–20. doi: 10.1111/nmo.12295 24372793

[38] Hsiao EY McBride SW Hsien S Sharon G Hyde ER McCue T. Microbiota Modulate Behavioral and Physiological Abnormalities Associated With Neurodevelopmental Disorders. Cell (2013) 155:1451–63. doi: 10.1016/j.cell.2013.11.024 PMC3897394 24315484

[39] Grochowska M Wojnar M Radkowski M. The Gut Microbiota in Neuropsychiatric Disorders. Acta Neurobiologiae Exp (2018) 78:69–81. doi: 10.21307/ane-2018-008 30019700

[40] Huang C Li Y Feng X Li D Li X Ouyang Q. Distinct Gut Microbiota Composition and Functional Category in Children With Cerebral Palsy and Epilepsy. Front Pediatr (2019) 7:394. doi: 10.3389/fped.2019.00394 31646147 PMC6779726

[41] Kovatcheva-Datchary P Nilsson A Akrami R Lee YS De Vadder F Arora T. Dietary Fiber-Induced Improvement in Glucose Metabolism Is Associated With Increased Abundance of Prevotella. Cell Metab (2015) 22:971–82. doi: 10.1016/j.cmet.2015.10.001 26552345

[42] Randis TM Ratner AJ. Gardnerella and Prevotella: Co-Conspirators in the Pathogenesis of Bacterial Vaginosis. J Infect Dis (2019) 220:1085–8. doi: 10.1093/infdis/jiy705 PMC6736359 30715397

[43] Arweiler NB Netuschil L. The Oral Microbiota. Adv Exp Med Biol (2016) 902:45–60. doi: 10.1007/978-3-319-31248-4_4 27161350

[44] Iljazovic A Roy U Gálvez EJC Lesker TR Zhao B Gronow A. Perturbation of the Gut Microbiome by Prevotella Spp. Enhances Host Susceptibility to Mucosal Inflammation. Mucosal Immunol (2021) 14:113–24. doi: 10.1038/s41385-020-0296-4 PMC7790746 32433514

[45] Kühn F Adiliaghdam F Cavallaro PM Hamarneh SR Tsurumi A Hoda RS. Intestinal Alkaline Phosphatase Targets the Gut Barrier to Prevent Aging. JCI Insight (2020) 5:134049. doi: 10.1172/jci.insight.134049 32213701 PMC7213802

