# Supplementary figures and images for: Evaluation of the Anti-Aging Effects of a Probiotic Combination Isolated From Centenarians in a SAMP8 Mouse Model
Source: Front Immunol. 2021 Dec 2;12:792746. doi: 10.3389/fimmu.2021.792746 (PMC8674427; doi:10.3389/fimmu.2021.792746)

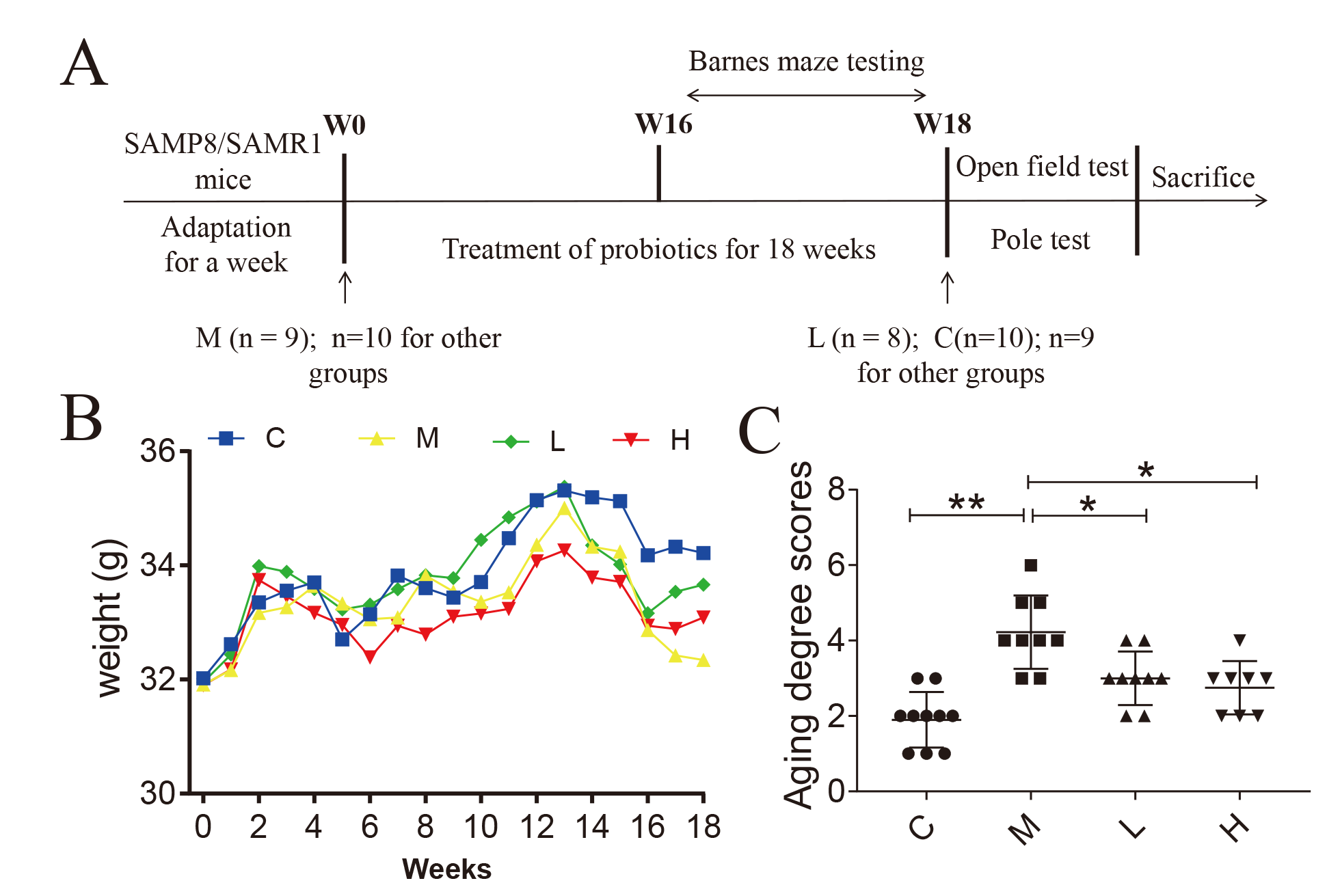

Supplement: Supplementary Figure 1 — The probiotic combination delayed the decrease in body weight and the increase in the aging score of SAMP8 mice. (A) The experimental design of this study. (B) Weekly body weight changes of SAMP8 mice during gavage of the probiotic combination. (C) The aging degree scores of SAMP8 mice after gavage of the probiotic combination. C: control group (n = 10), M: model group (n = 9), L: how-dose probiotics group (n = 8), H: high-dose probiotics group (n = 9). Data are presented as the means ± SD. *p < 0.05 **p < 0.0. [file Image_1.tif]

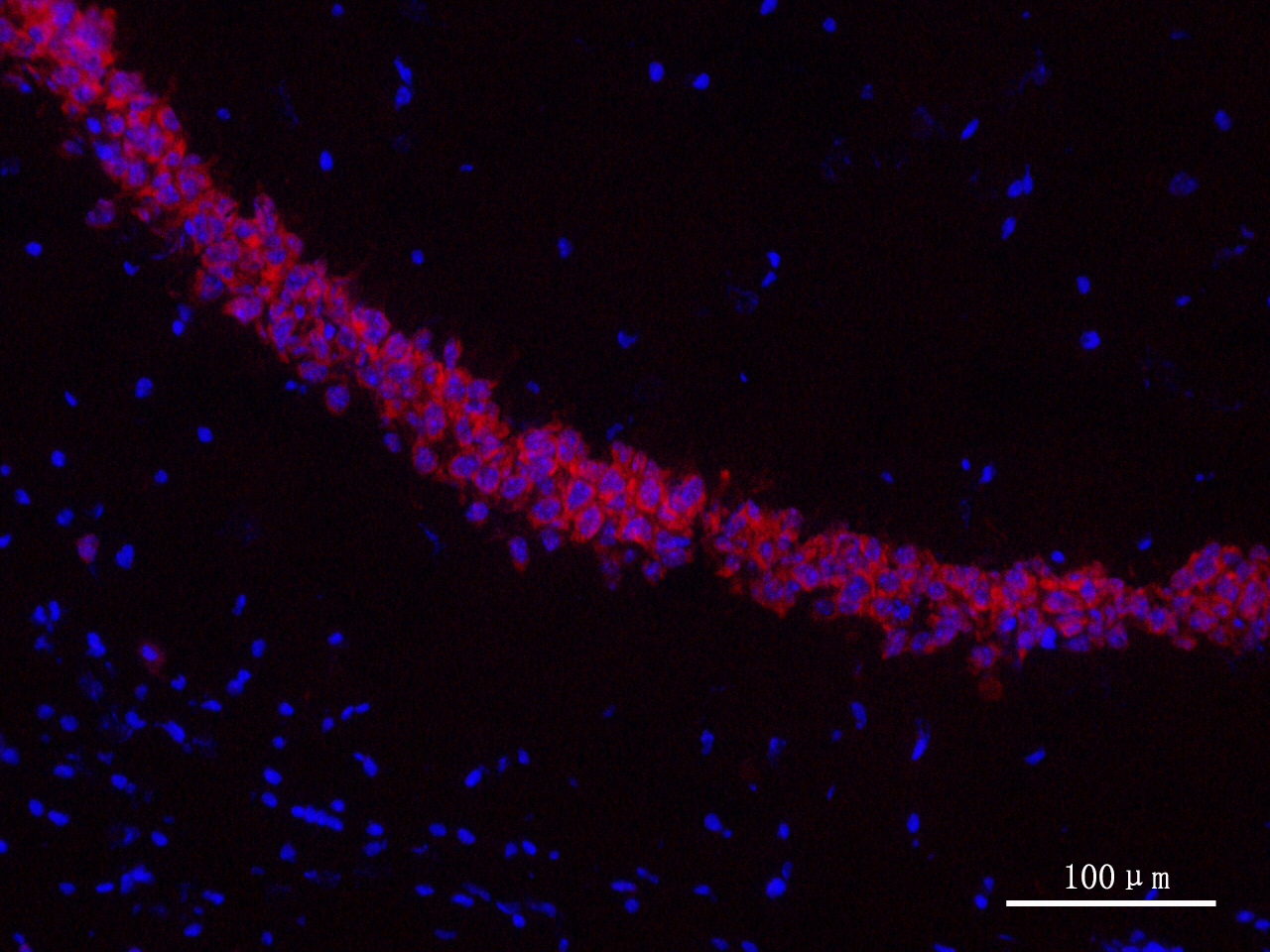

Supplement: Supplementary file 2 [file DataSheet_1.zip › original data/Figure 3/Figure 3A/C NEUNú¿║∞ú⌐ 200-3 4.jpg]

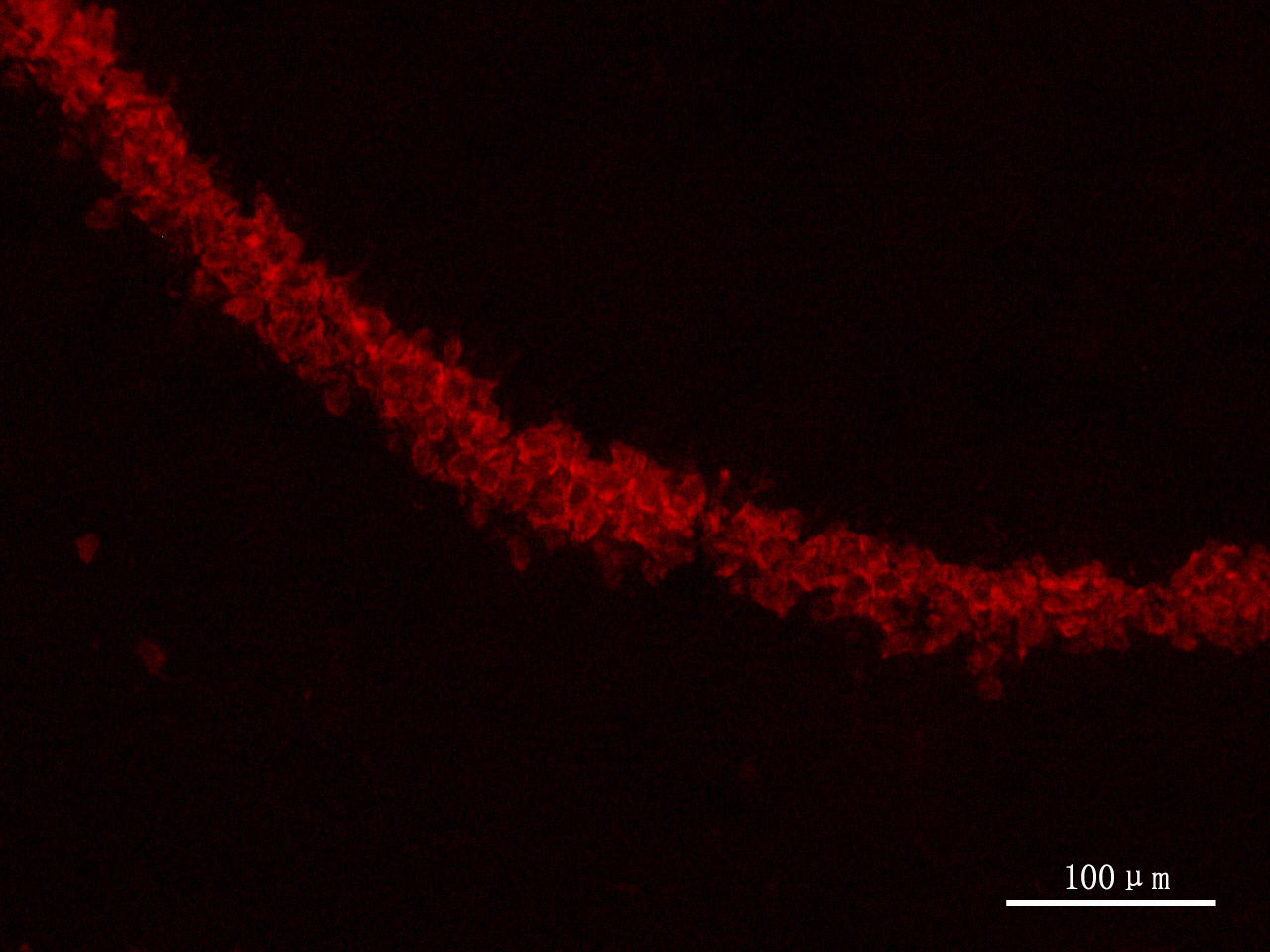

Supplement: Supplementary file 2 [file DataSheet_1.zip › original data/Figure 3/Figure 3A/C NEUNú¿║∞ú⌐ 200-3.jpg]

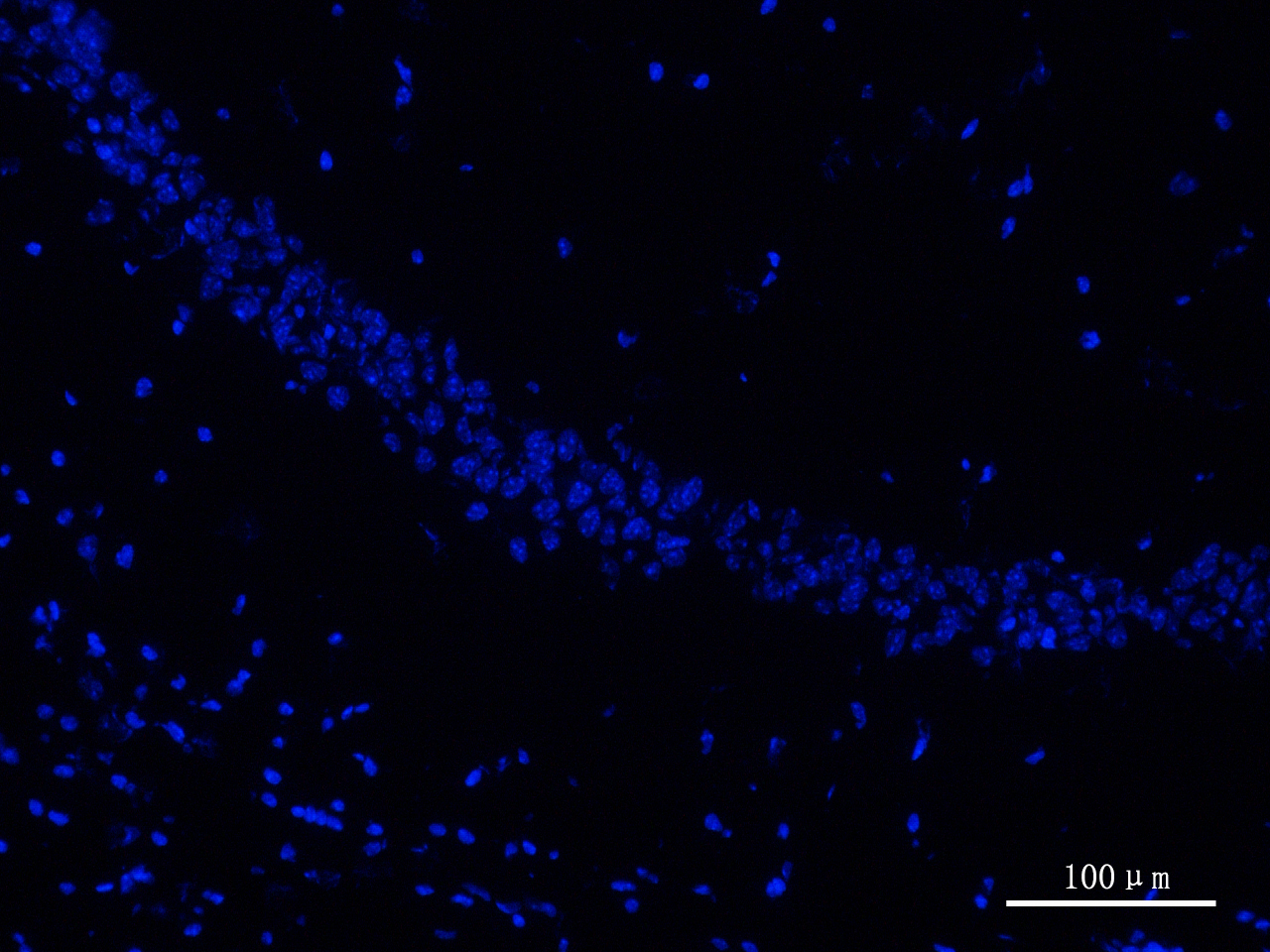

Supplement: Supplementary file 2 [file DataSheet_1.zip › original data/Figure 3/Figure 3A/C NEUNú¿║∞ú⌐ 200-4.jpg]

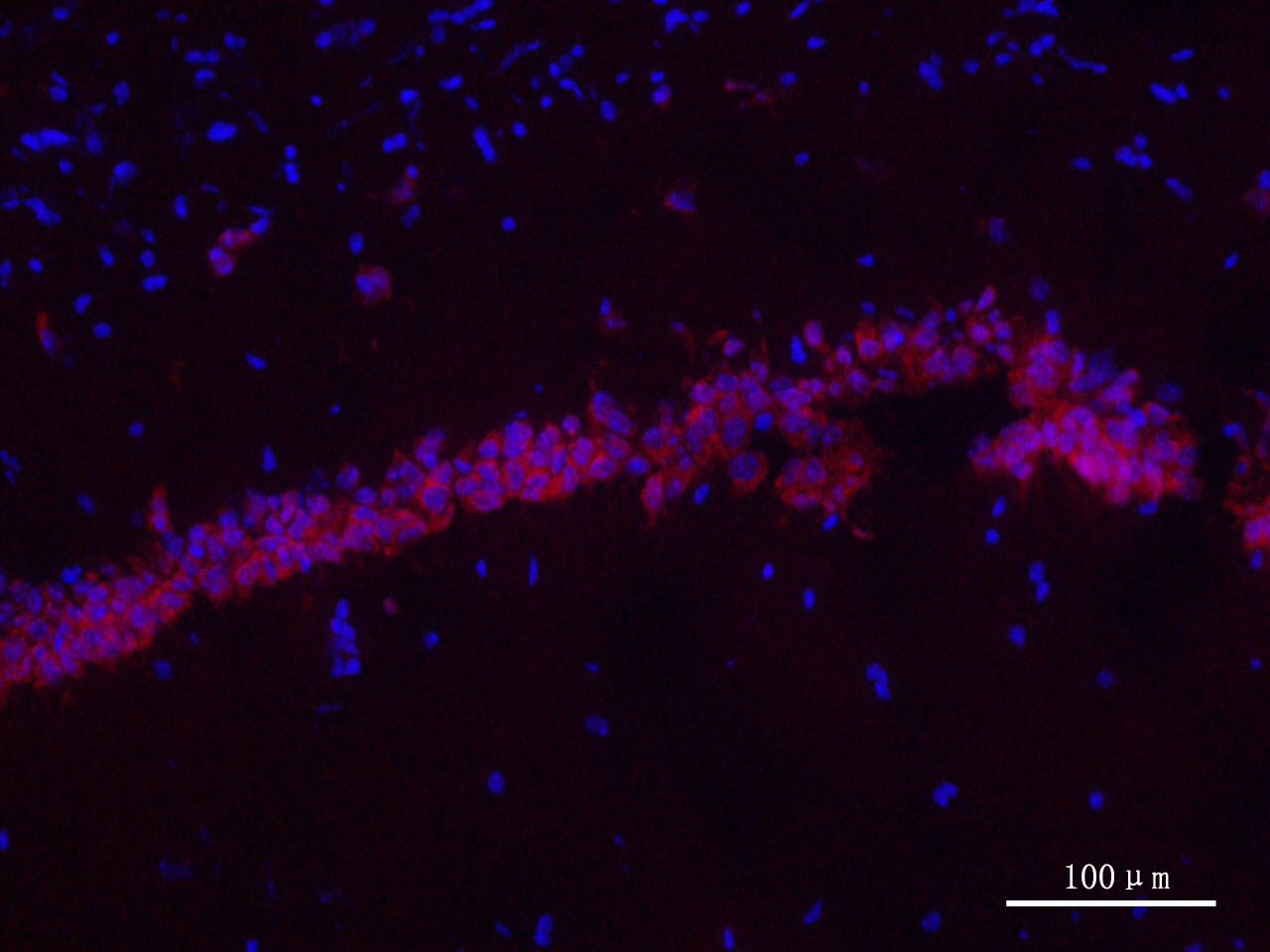

Supplement: Supplementary file 2 [file DataSheet_1.zip › original data/Figure 3/Figure 3A/H NEUNú¿║∞ú⌐ 200-3 4.jpg]

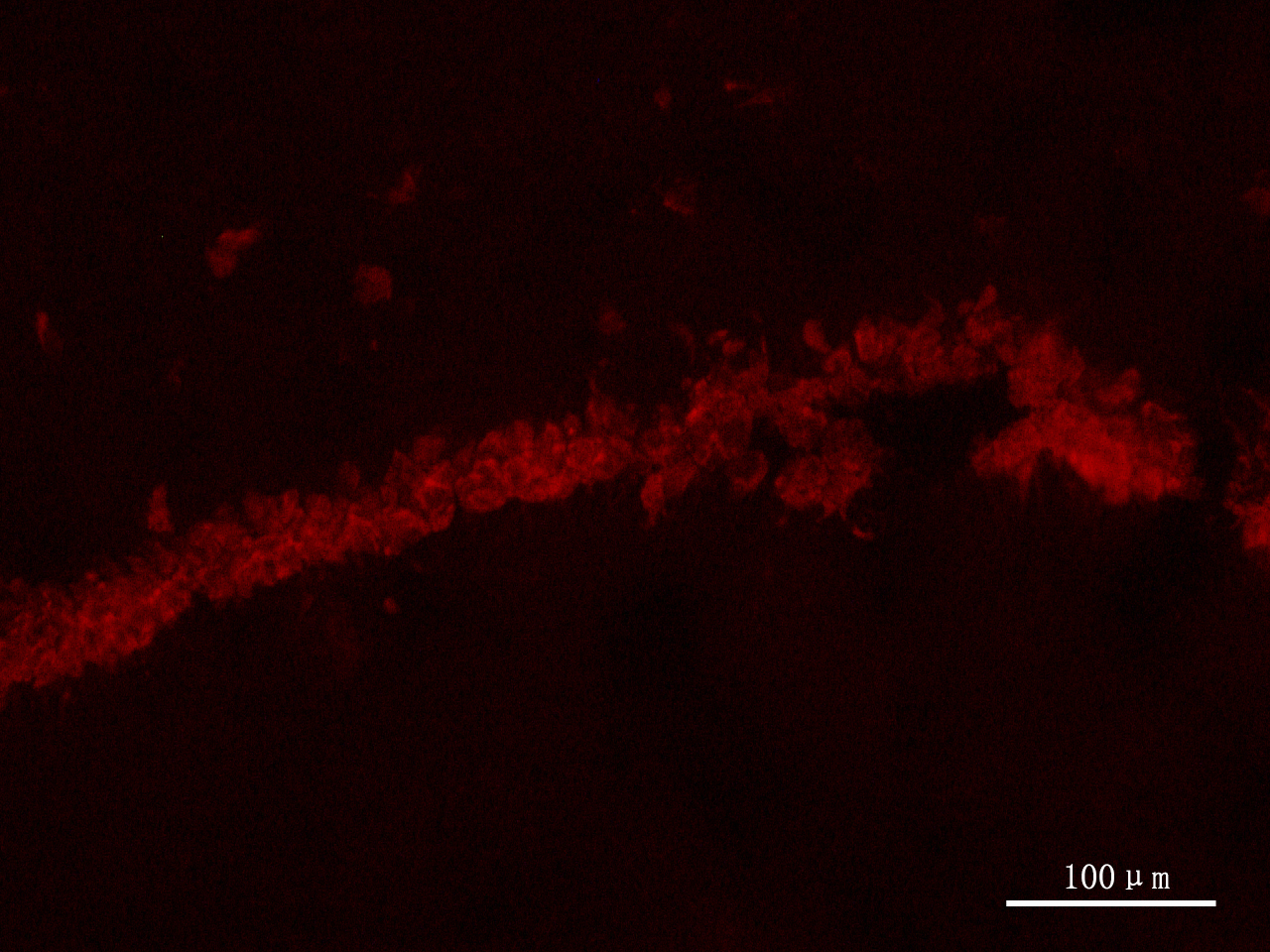

Supplement: Supplementary file 2 [file DataSheet_1.zip › original data/Figure 3/Figure 3A/H NEUNú¿║∞ú⌐ 200-3.jpg]

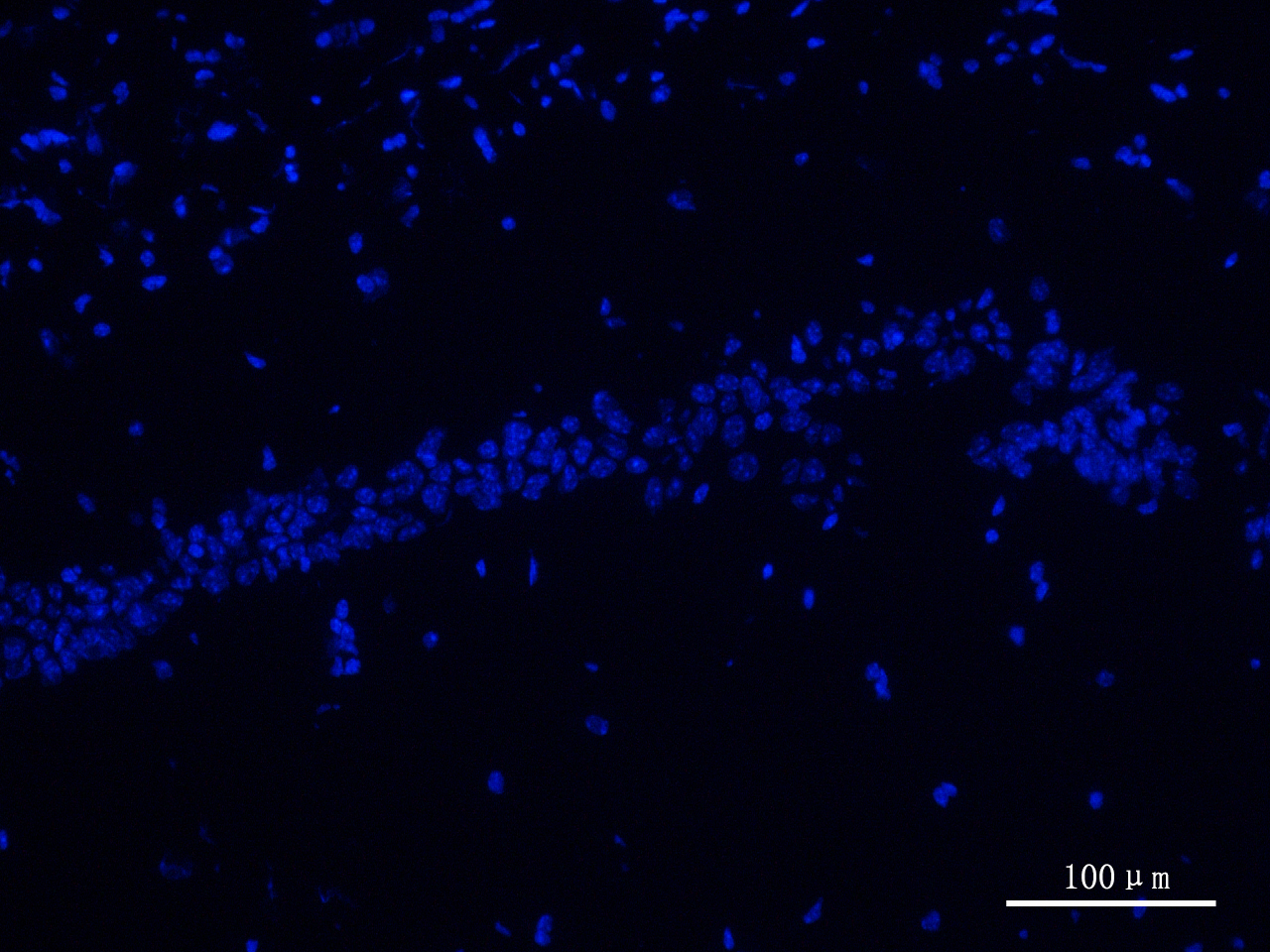

Supplement: Supplementary file 2 [file DataSheet_1.zip › original data/Figure 3/Figure 3A/H NEUNú¿║∞ú⌐ 200-4.jpg]

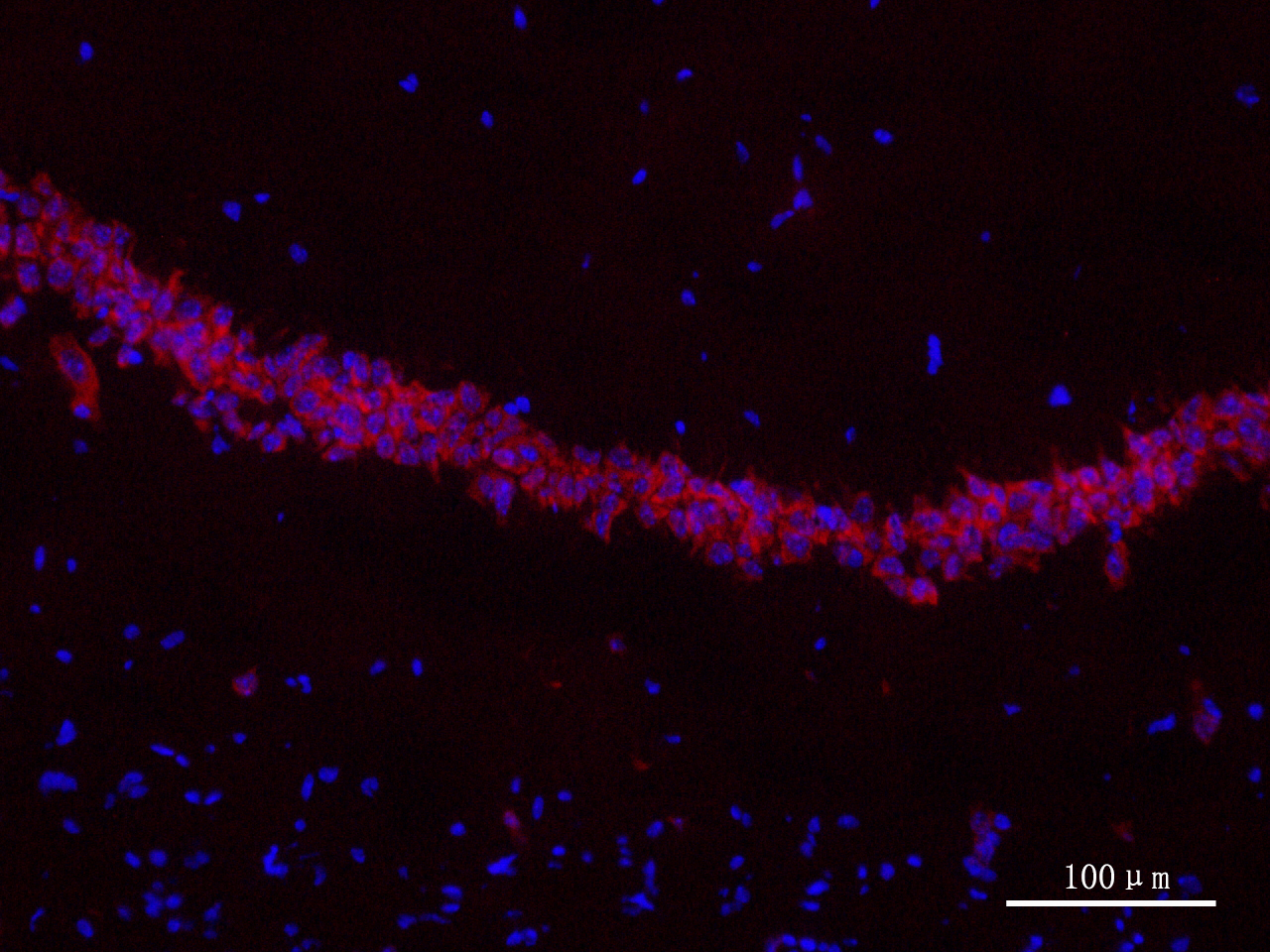

Supplement: Supplementary file 2 [file DataSheet_1.zip › original data/Figure 3/Figure 3A/L NEUNú¿║∞ú⌐ 200-3 4.jpg]

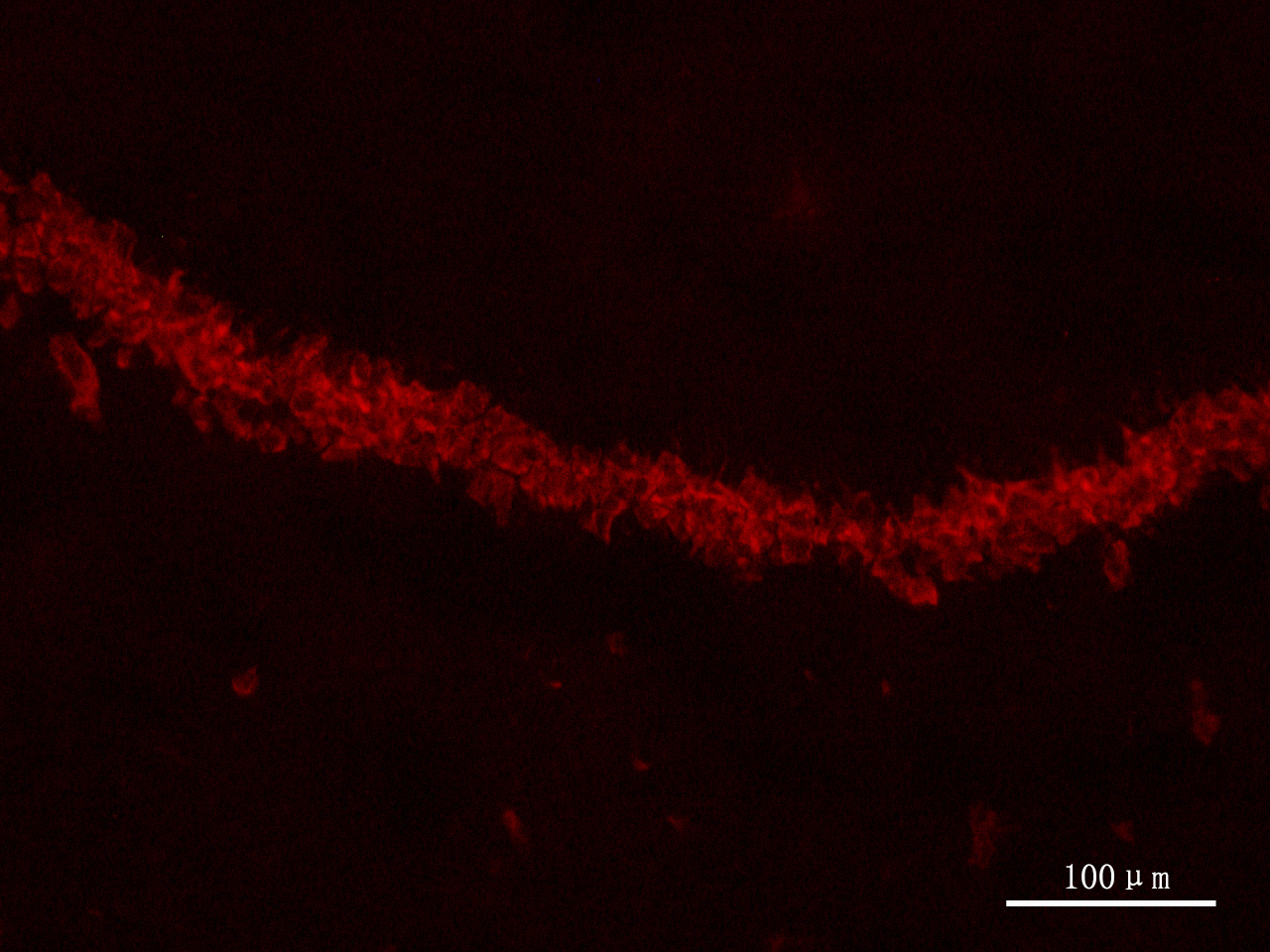

Supplement: Supplementary file 2 [file DataSheet_1.zip › original data/Figure 3/Figure 3A/L NEUNú¿║∞ú⌐ 200-3.jpg]

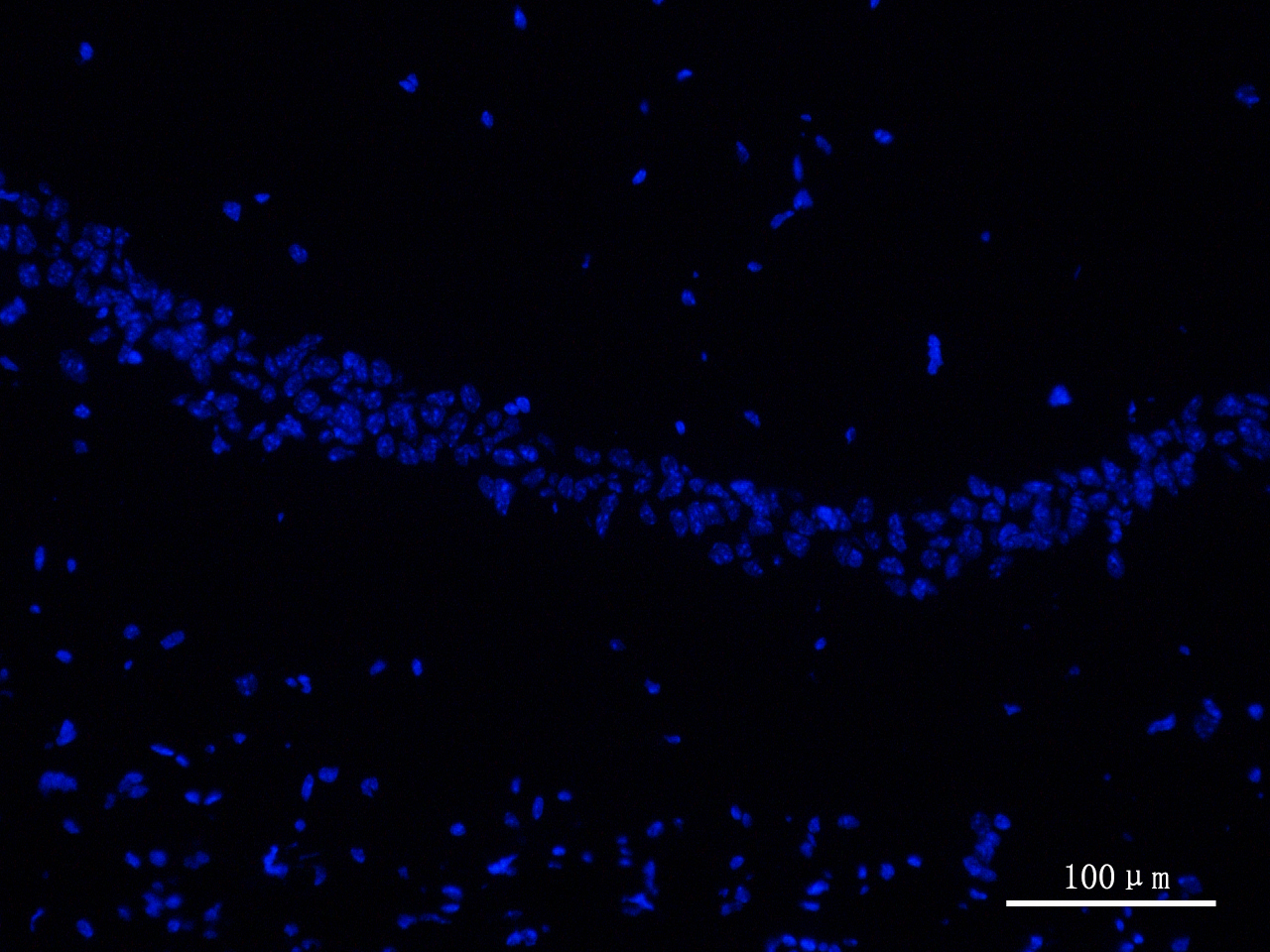

Supplement: Supplementary file 2 [file DataSheet_1.zip › original data/Figure 3/Figure 3A/L NEUNú¿║∞ú⌐ 200-4.jpg]

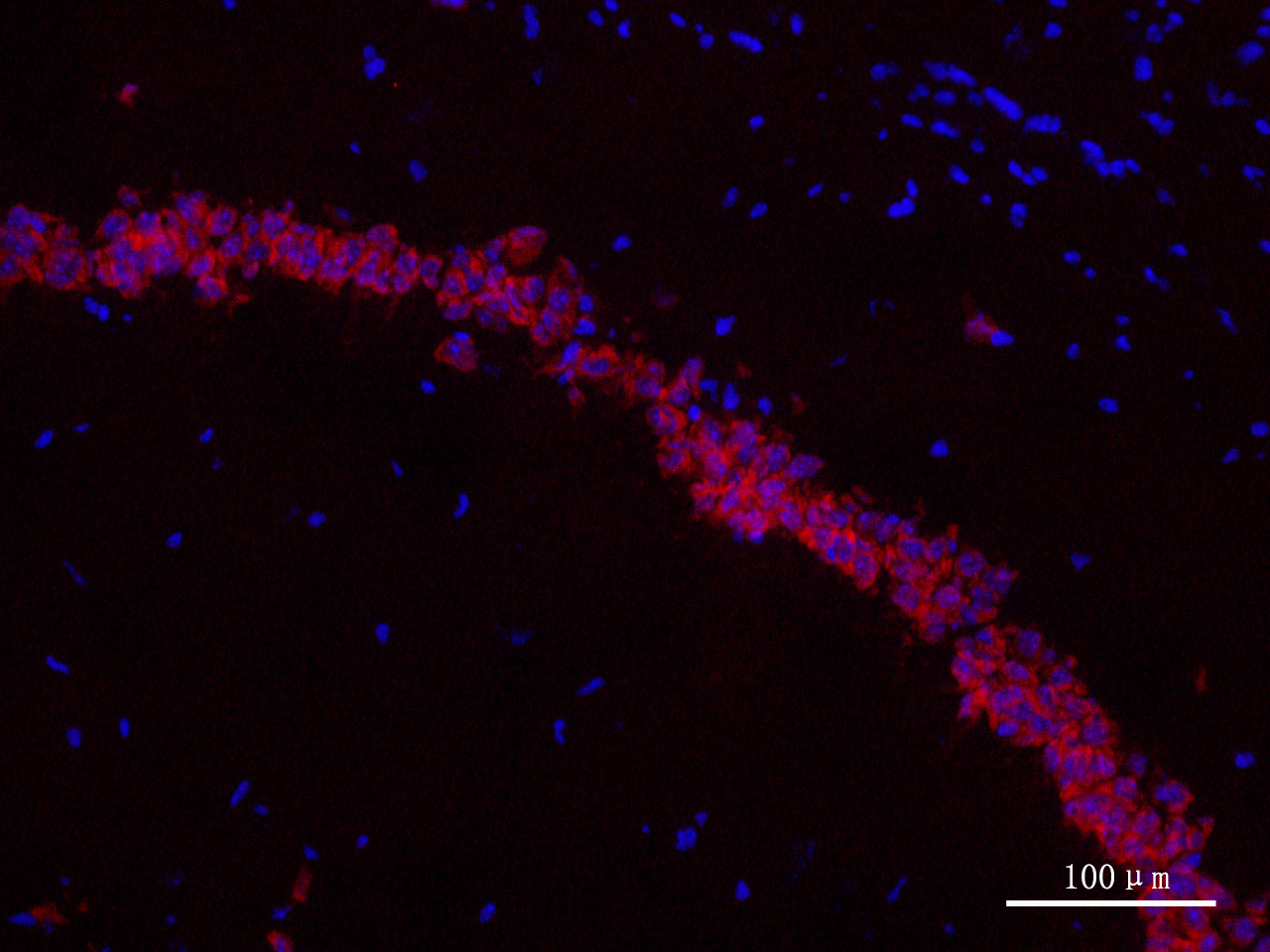

Supplement: Supplementary file 2 [file DataSheet_1.zip › original data/Figure 3/Figure 3A/M NEUNú¿║∞ú⌐ 200-3 4.jpg]

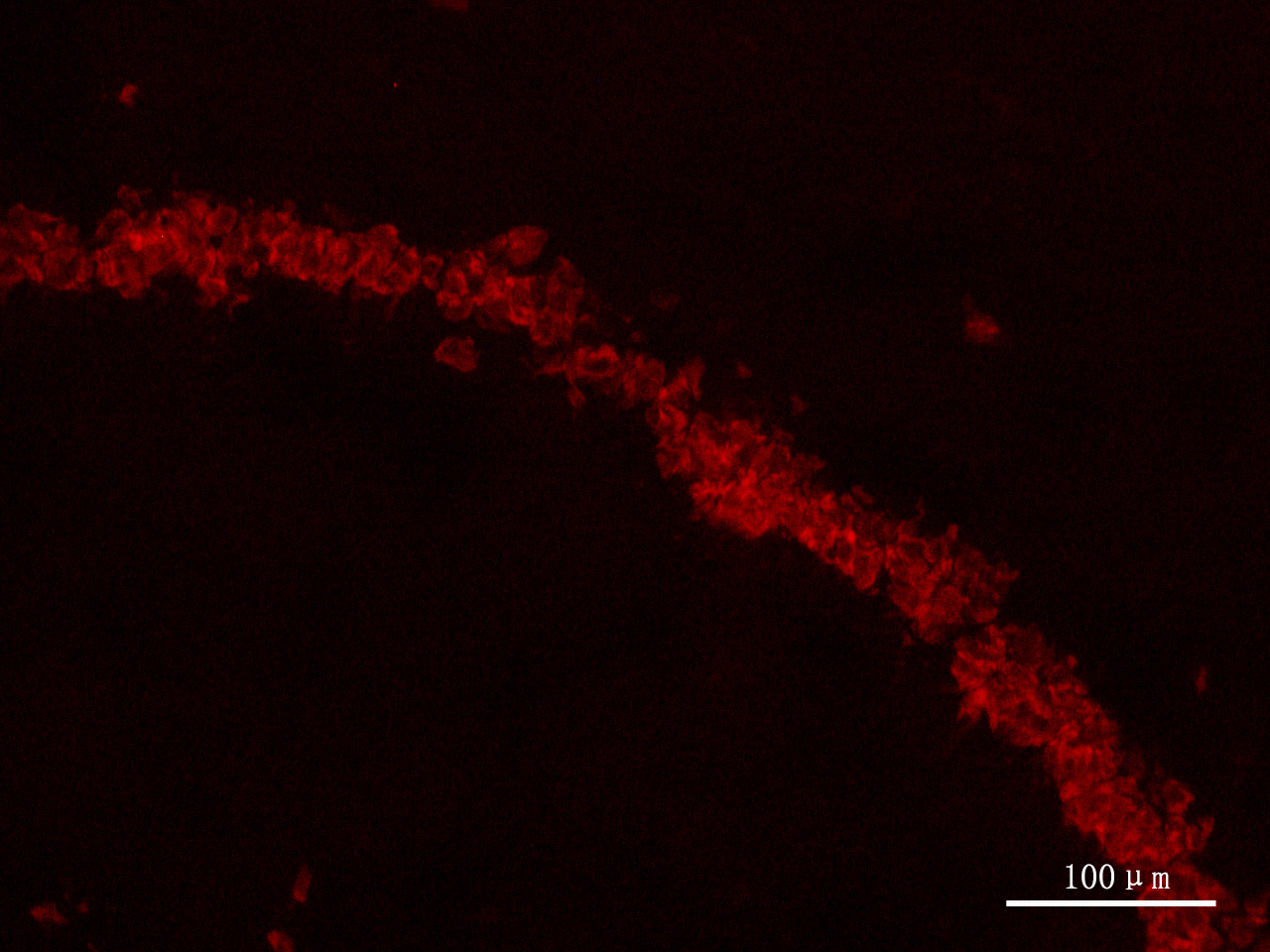

Supplement: Supplementary file 2 [file DataSheet_1.zip › original data/Figure 3/Figure 3A/M NEUNú¿║∞ú⌐ 200-3.jpg]

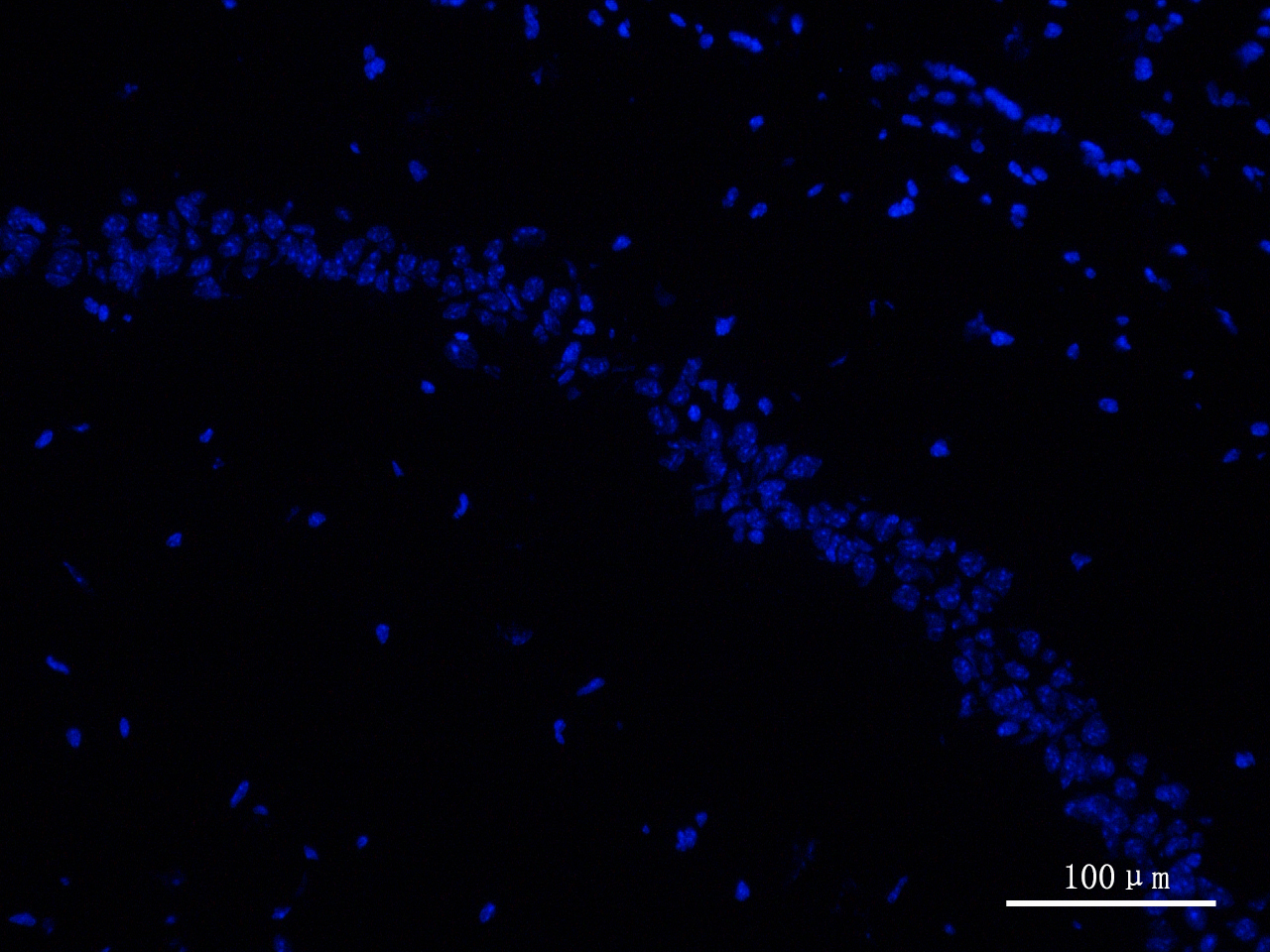

Supplement: Supplementary file 2 [file DataSheet_1.zip › original data/Figure 3/Figure 3A/M NEUNú¿║∞ú⌐ 200-4.jpg]

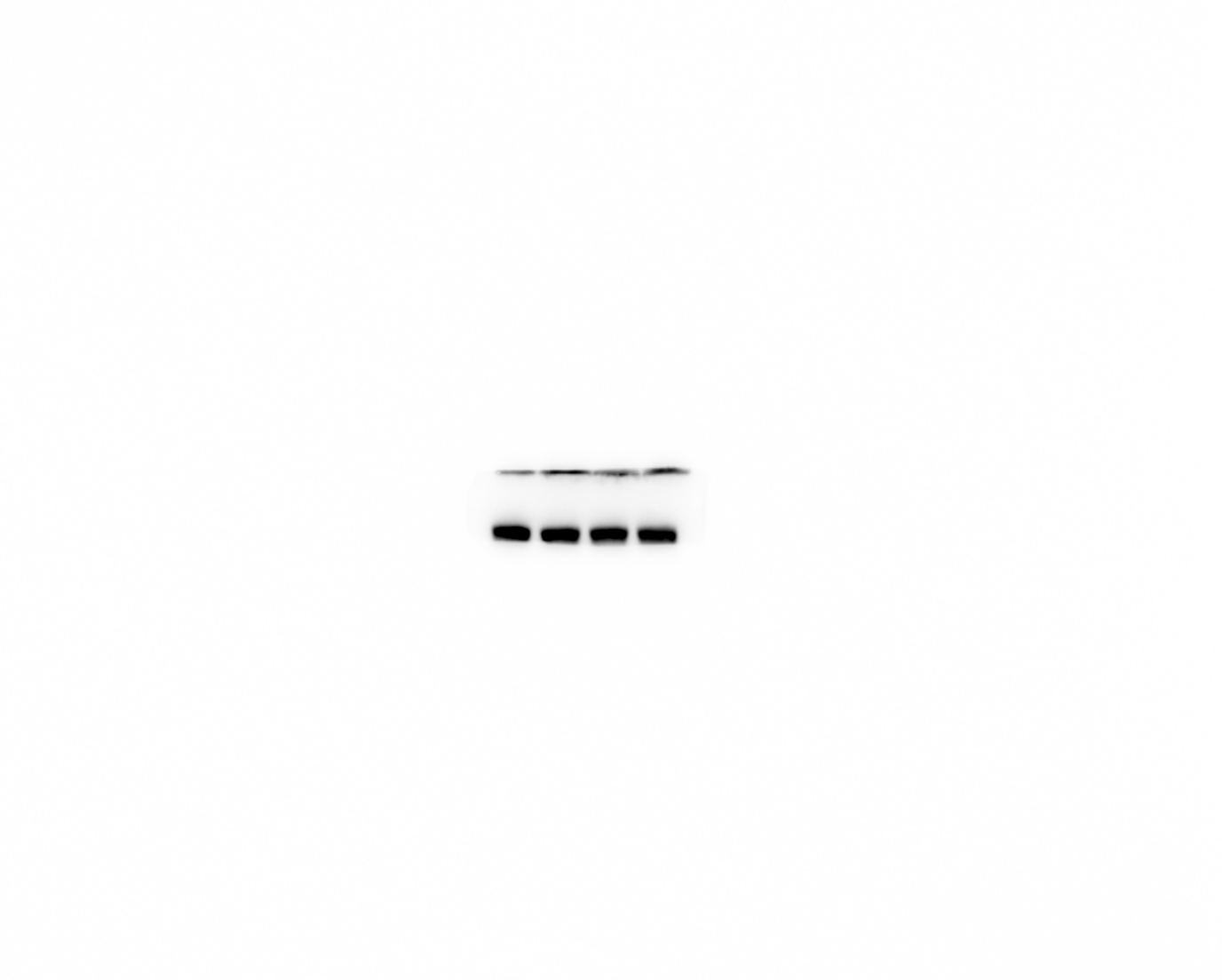

Supplement: Supplementary file 2 [file DataSheet_1.zip › original data/Figure 3/Figure 3B/AKT.Tif]

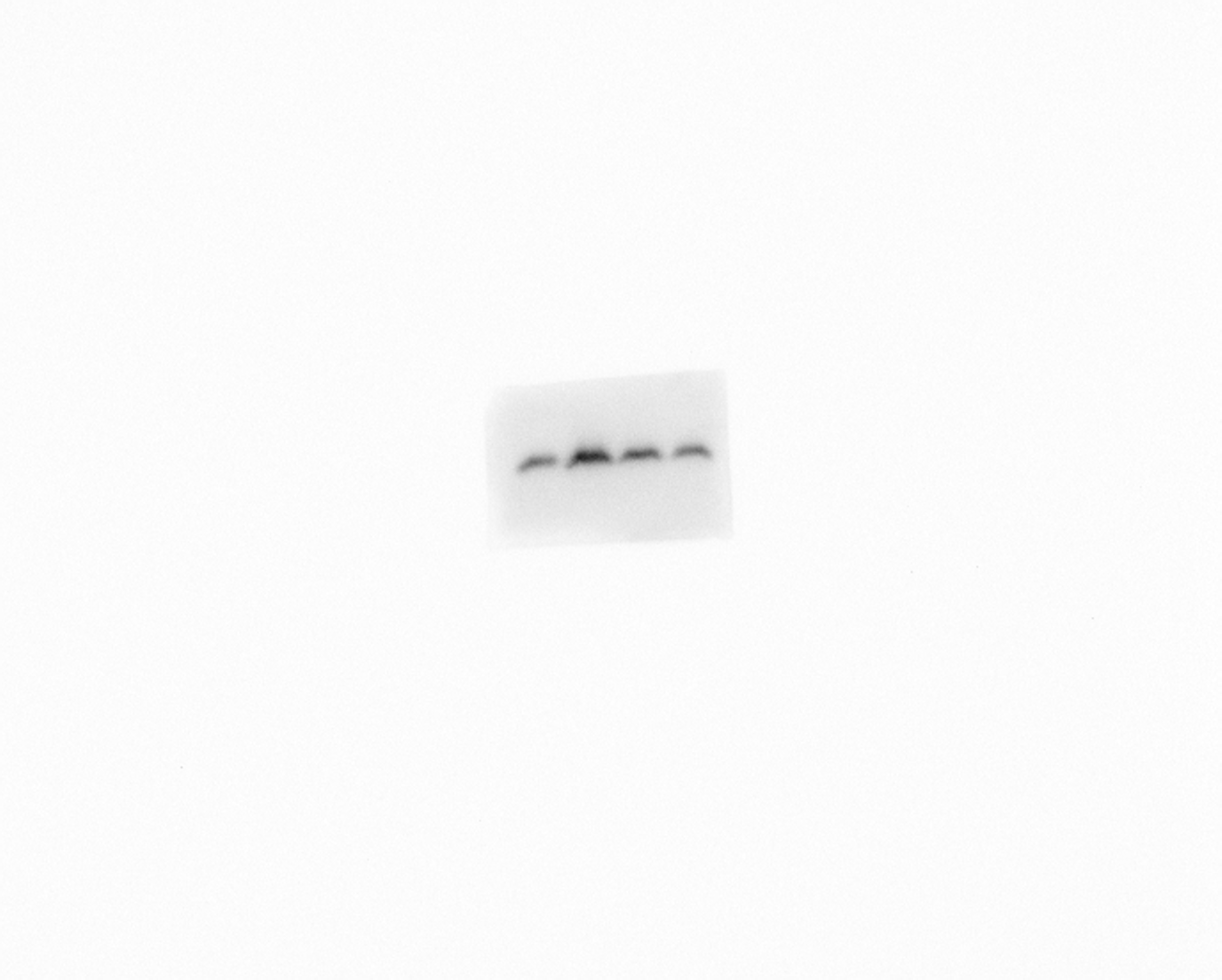

Supplement: Supplementary file 2 [file DataSheet_1.zip › original data/Figure 3/Figure 3B/BAX.Tif]

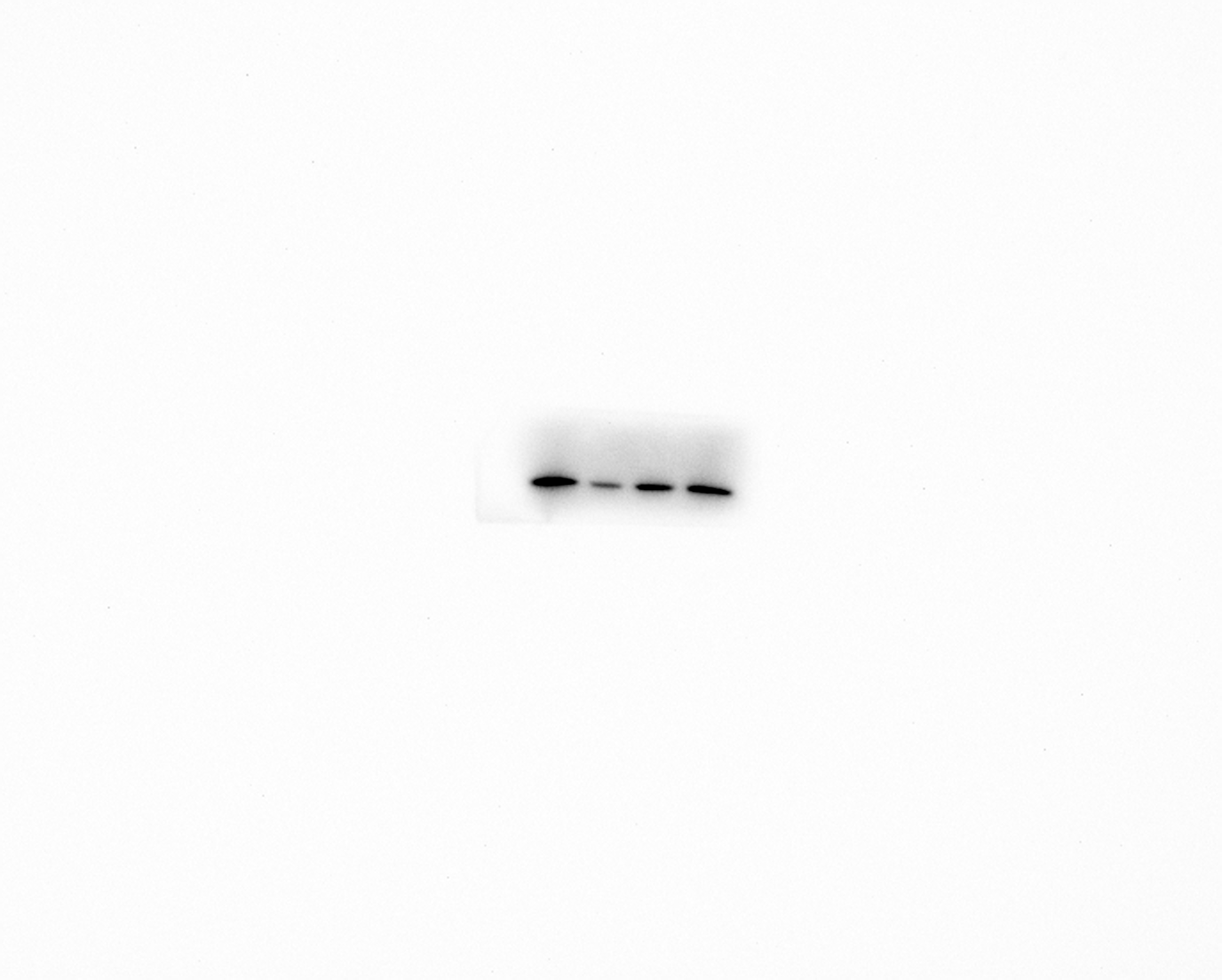

Supplement: Supplementary file 2 [file DataSheet_1.zip › original data/Figure 3/Figure 3B/BCL-2.Tif]

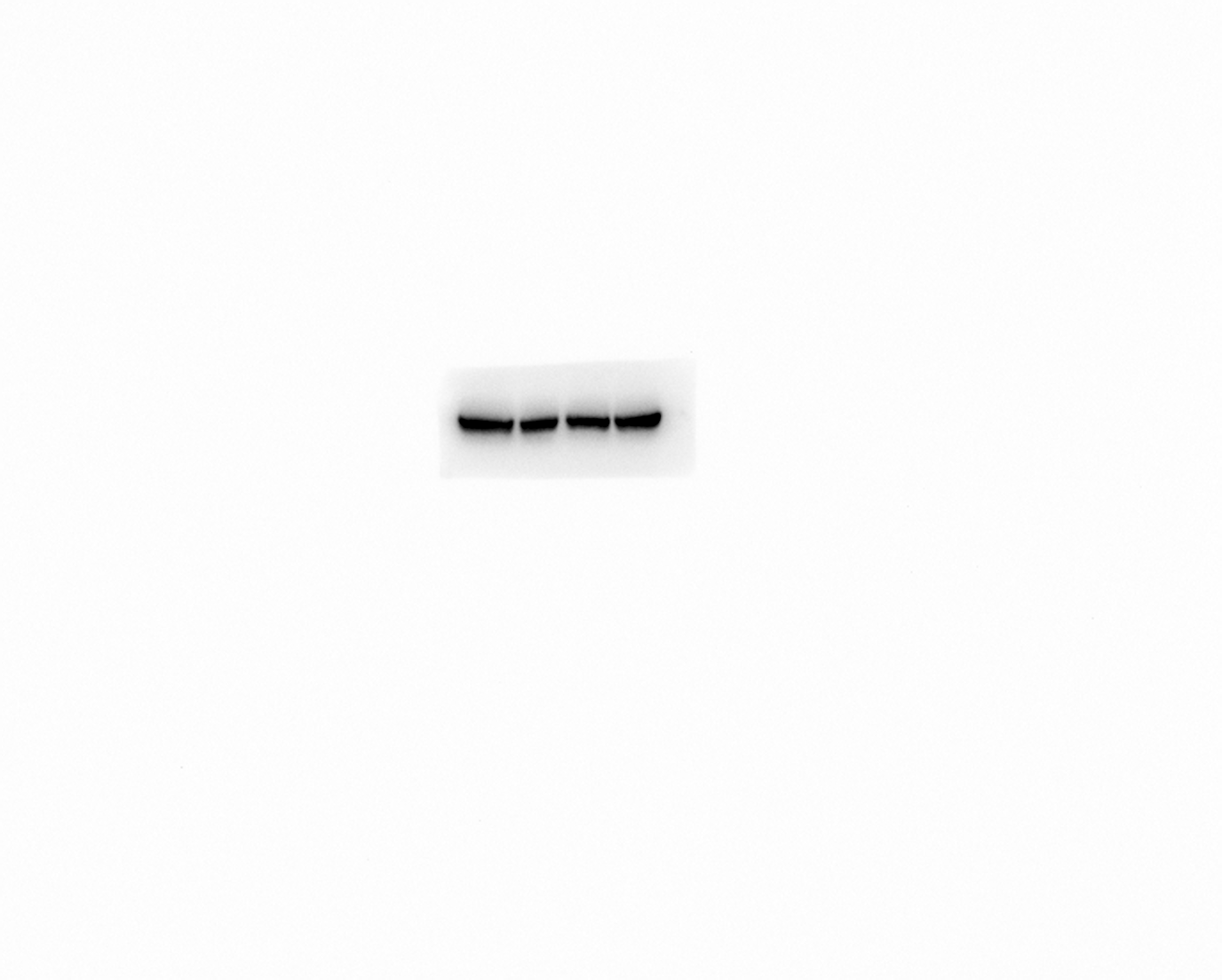

Supplement: Supplementary file 2 [file DataSheet_1.zip › original data/Figure 3/Figure 3B/pAKT.Tif]

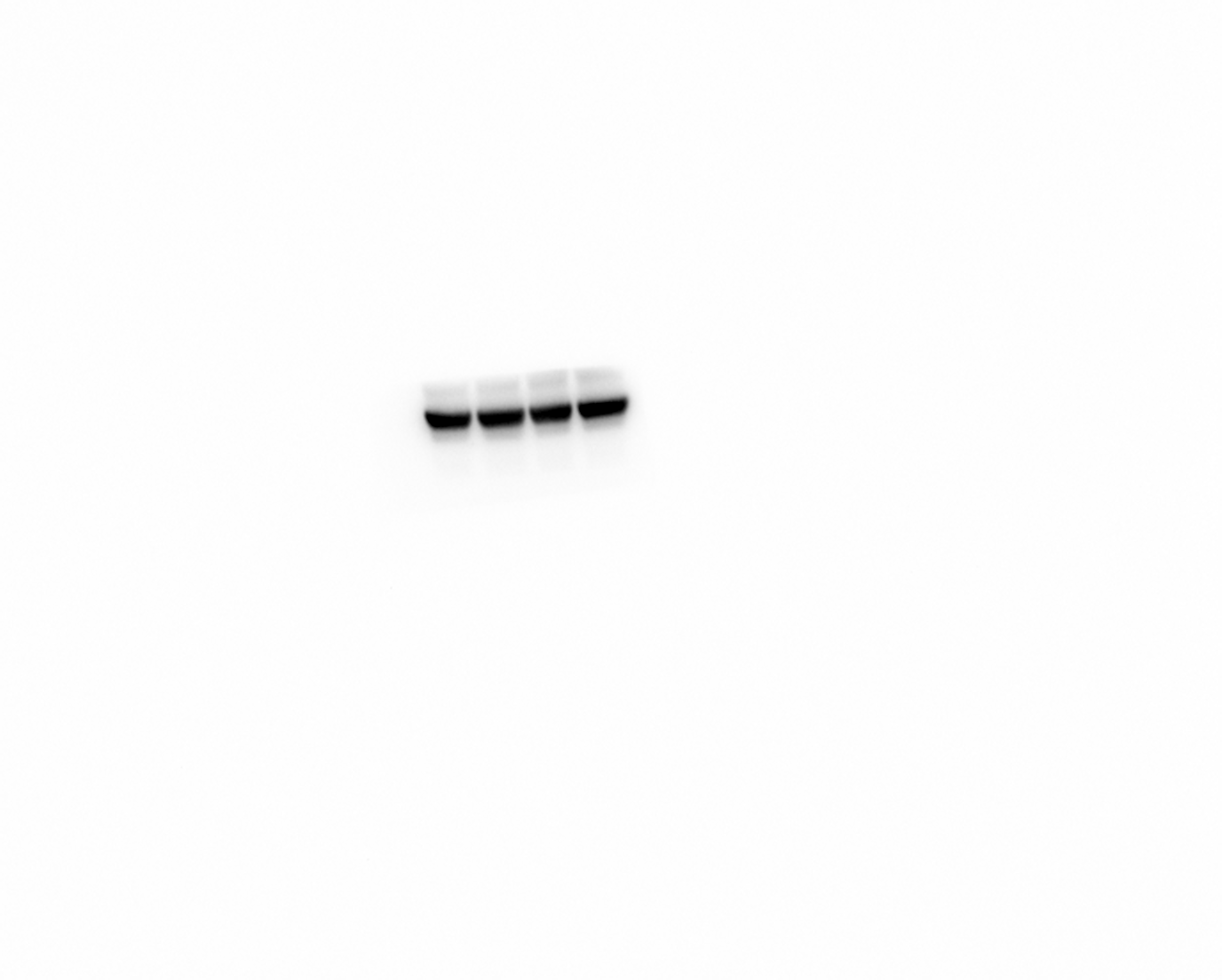

Supplement: Supplementary file 2 [file DataSheet_1.zip › original data/Figure 3/Figure 3B/a┬-ACTIN.Tif]

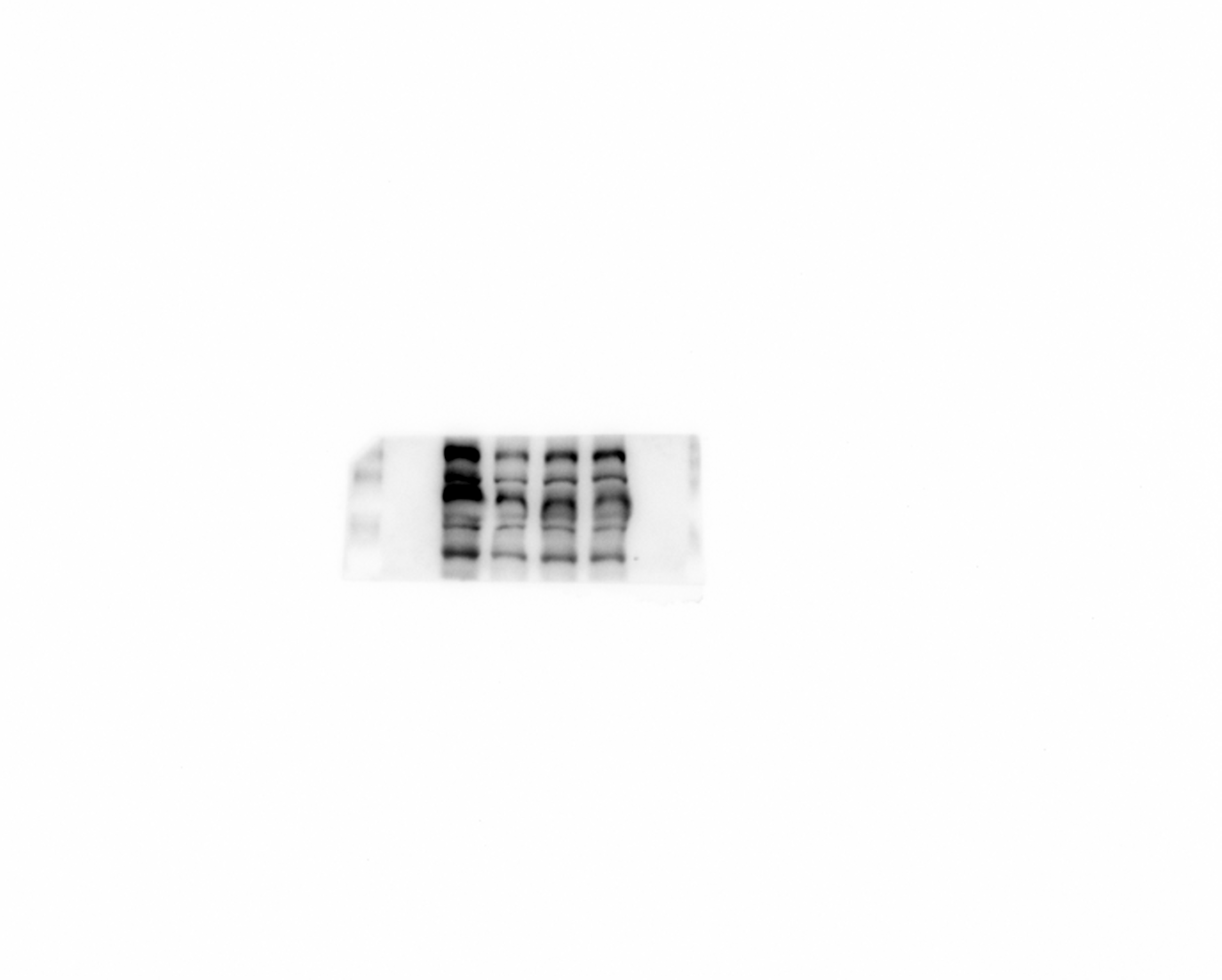

Supplement: Supplementary file 2 [file DataSheet_1.zip › original data/Figure 3/Figure 3C/SIRT1.Tif]

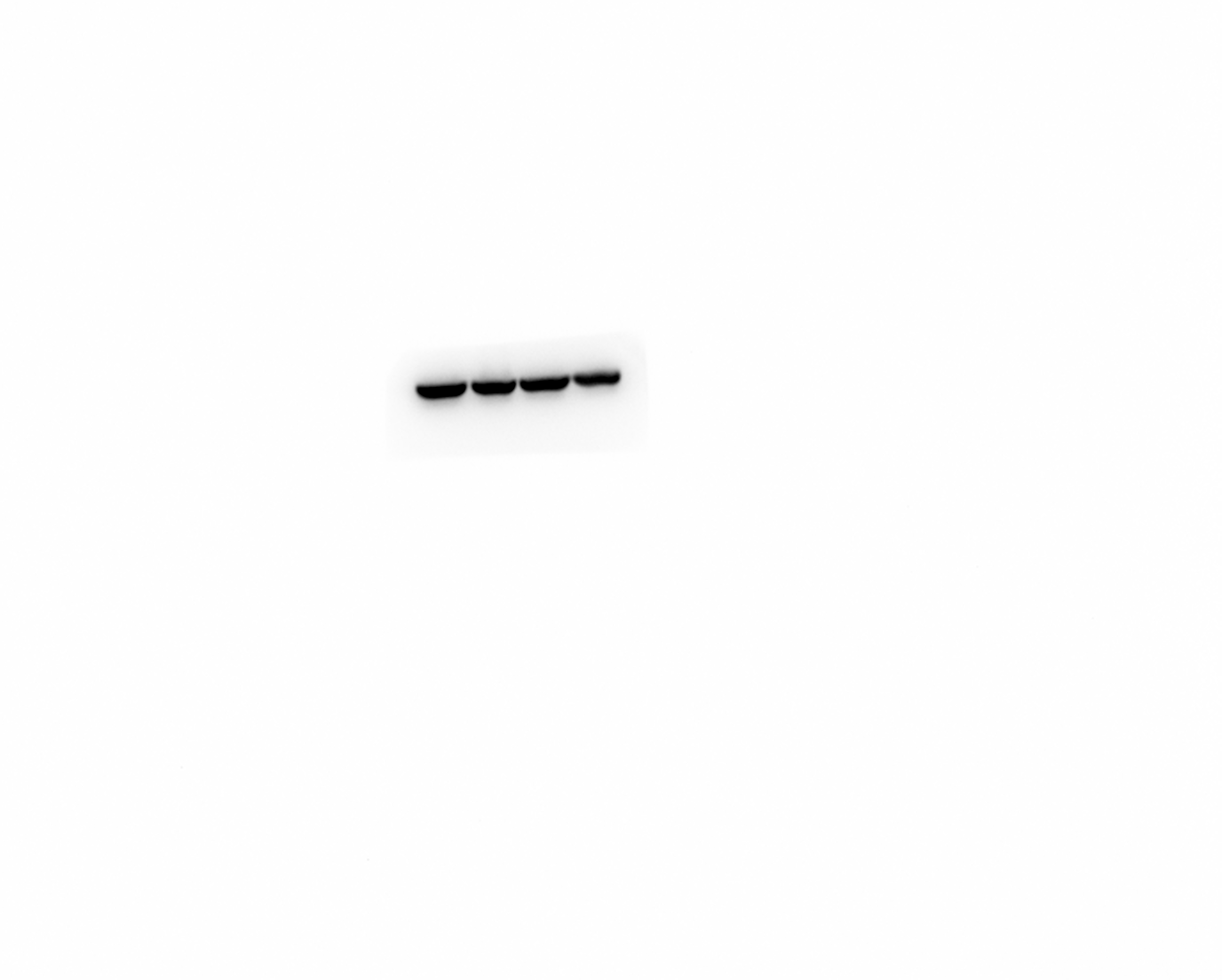

Supplement: Supplementary file 2 [file DataSheet_1.zip › original data/Figure 3/Figure 3C/a┬-ACTIN.Tif]

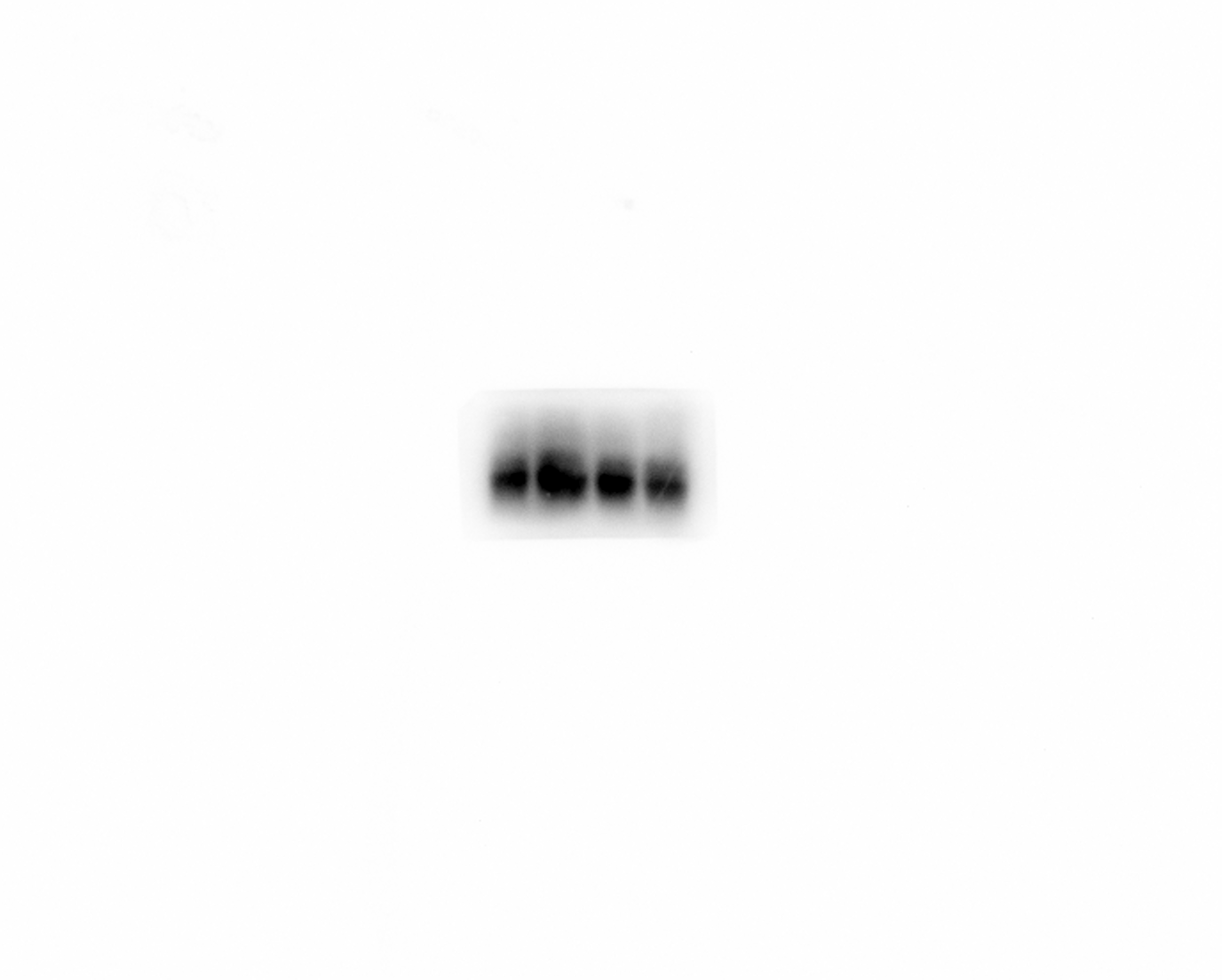

Supplement: Supplementary file 2 [file DataSheet_1.zip › original data/Figure 4/Figure 4A/MYD88.Tif]

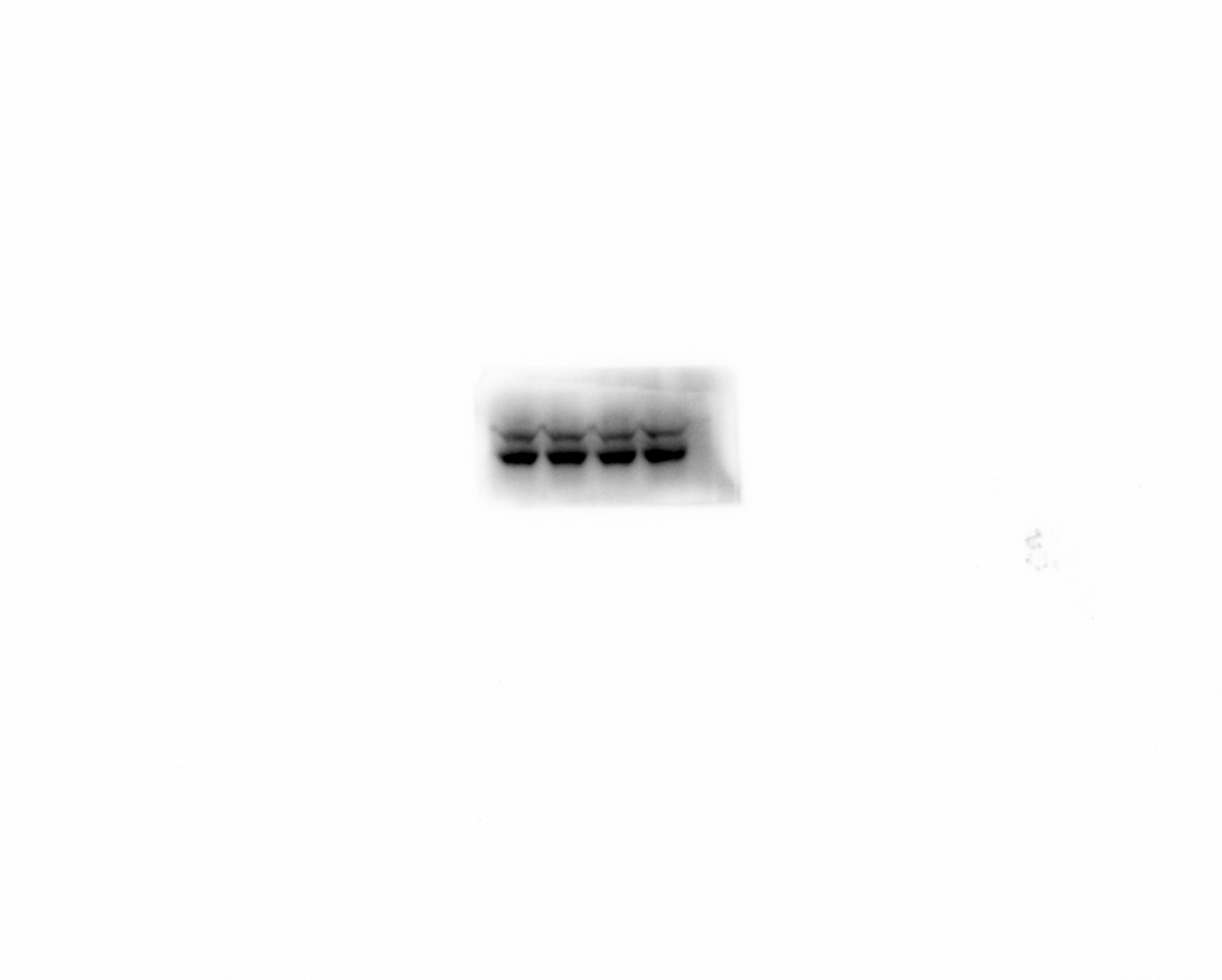

Supplement: Supplementary file 2 [file DataSheet_1.zip › original data/Figure 4/Figure 4A/P65.Tif]

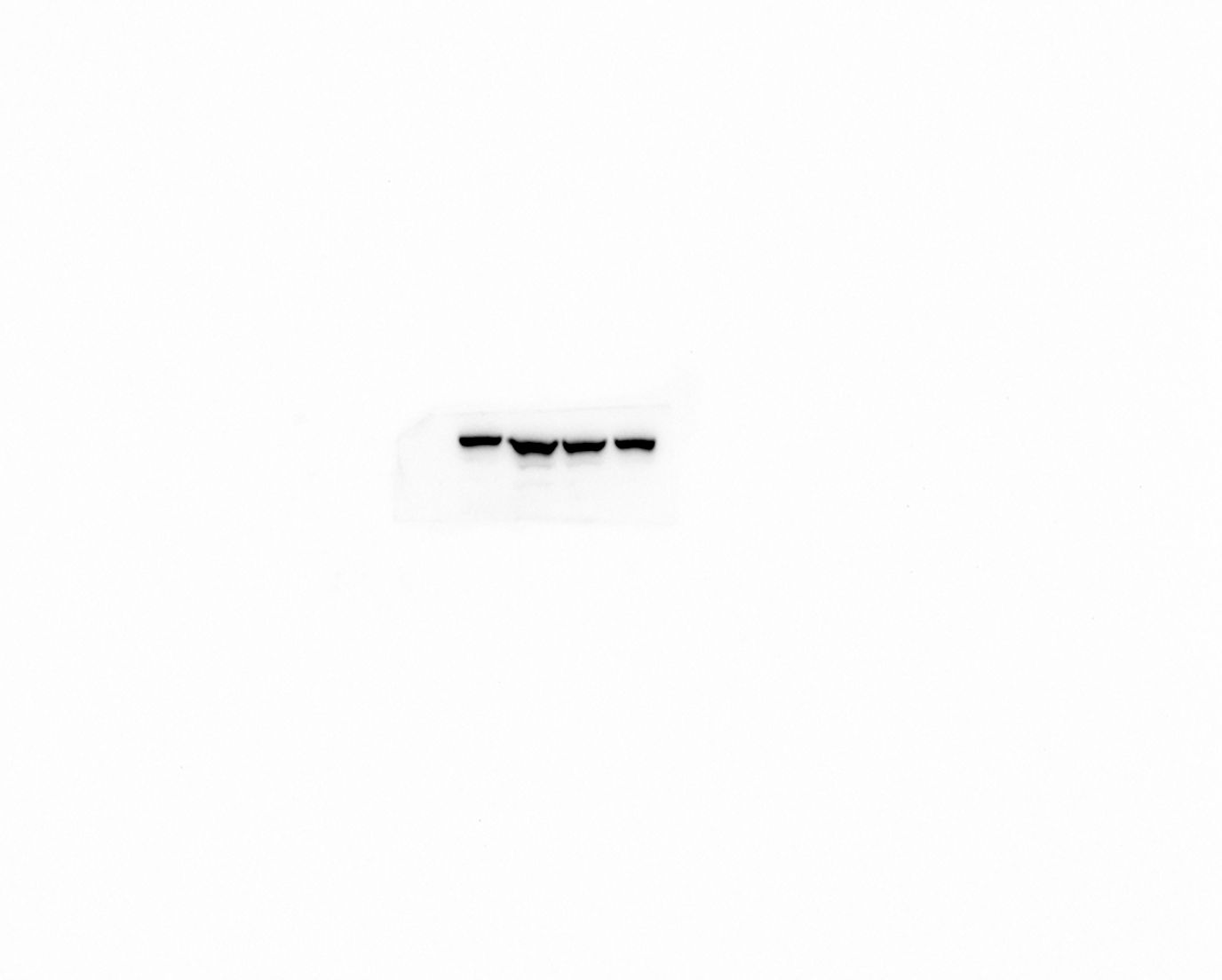

Supplement: Supplementary file 2 [file DataSheet_1.zip › original data/Figure 4/Figure 4A/TLR4.Tif]

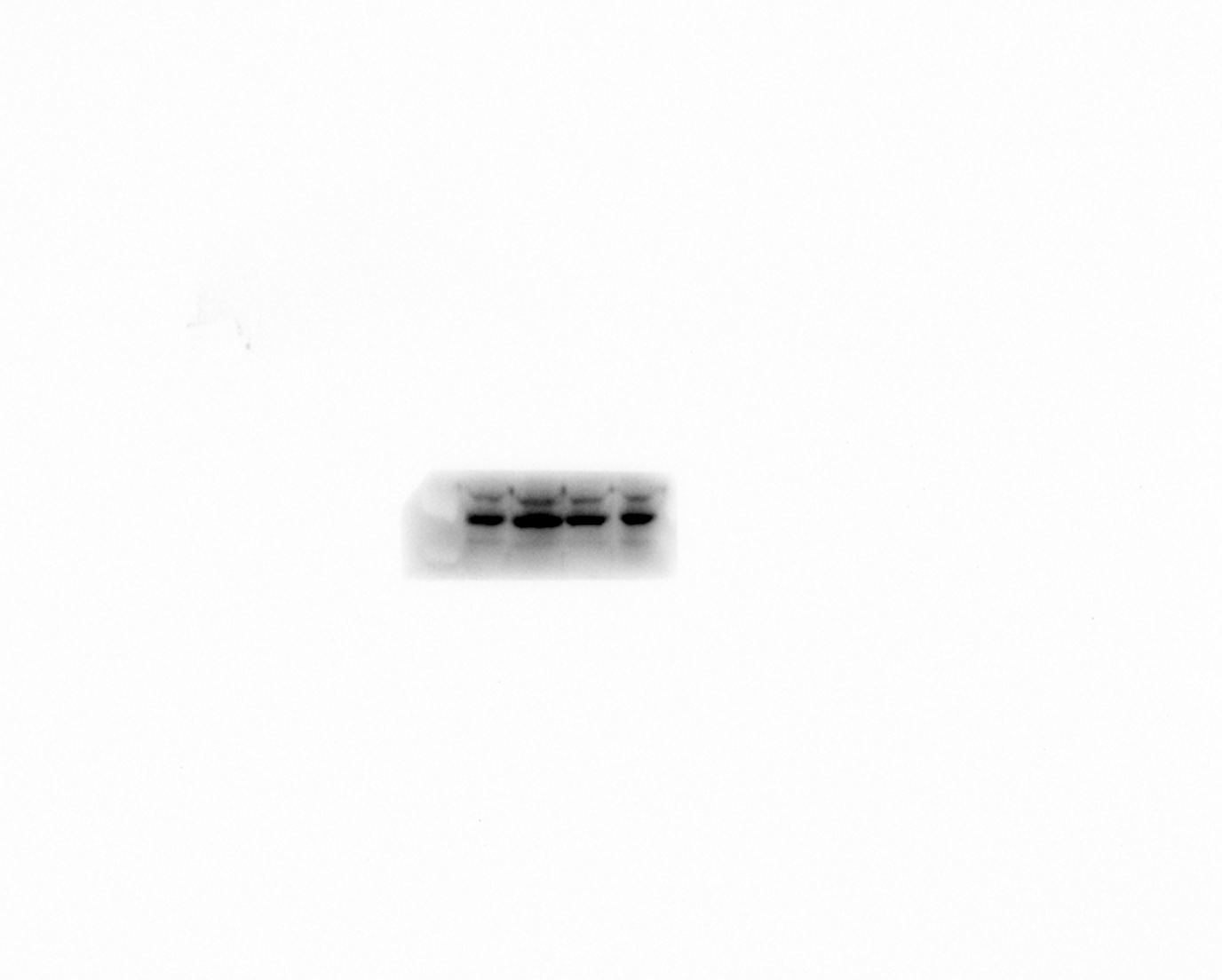

Supplement: Supplementary file 2 [file DataSheet_1.zip › original data/Figure 4/Figure 4A/pp65.Tif]

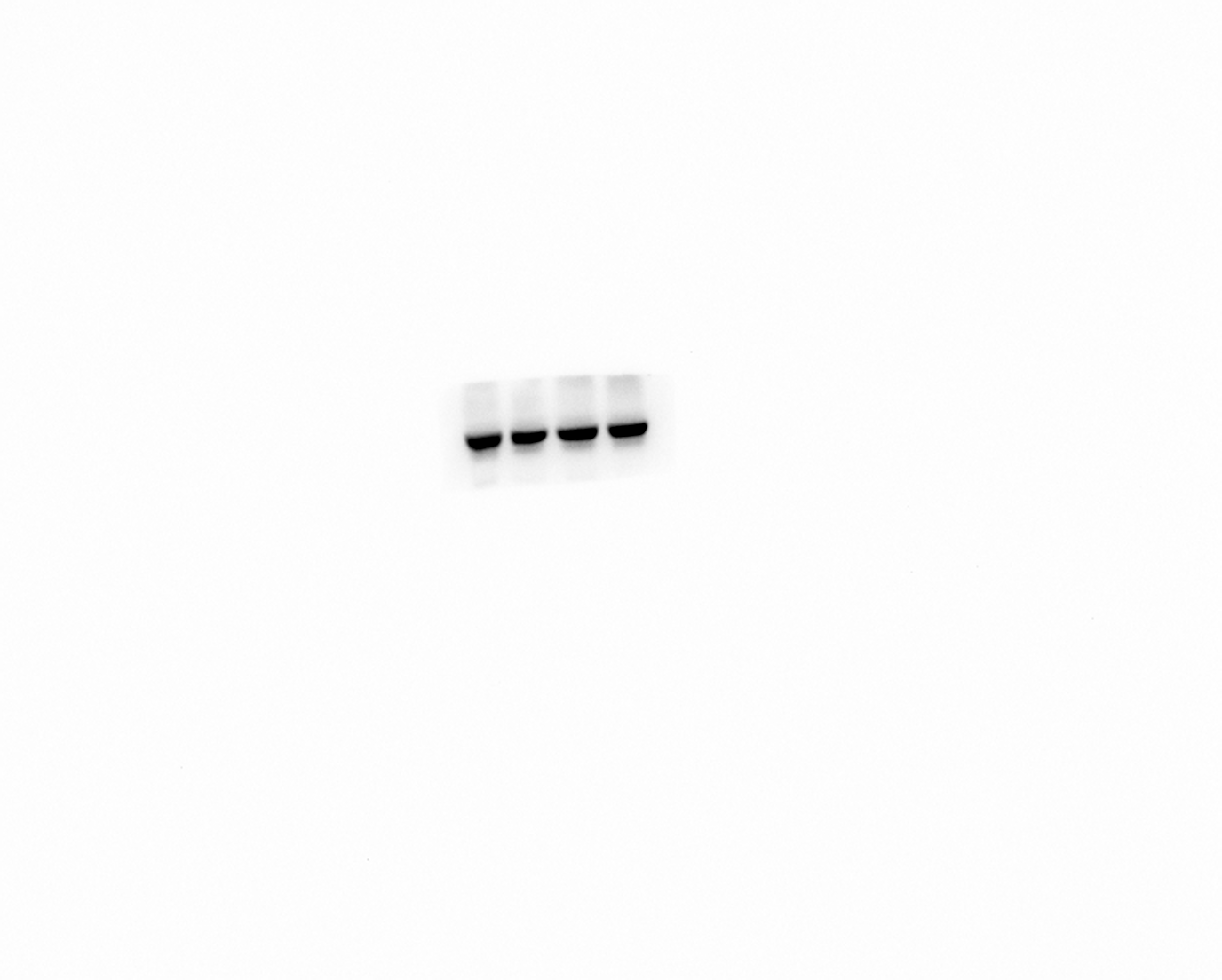

Supplement: Supplementary file 2 [file DataSheet_1.zip › original data/Figure 4/Figure 4A/a┬-ACTIN.Tif]

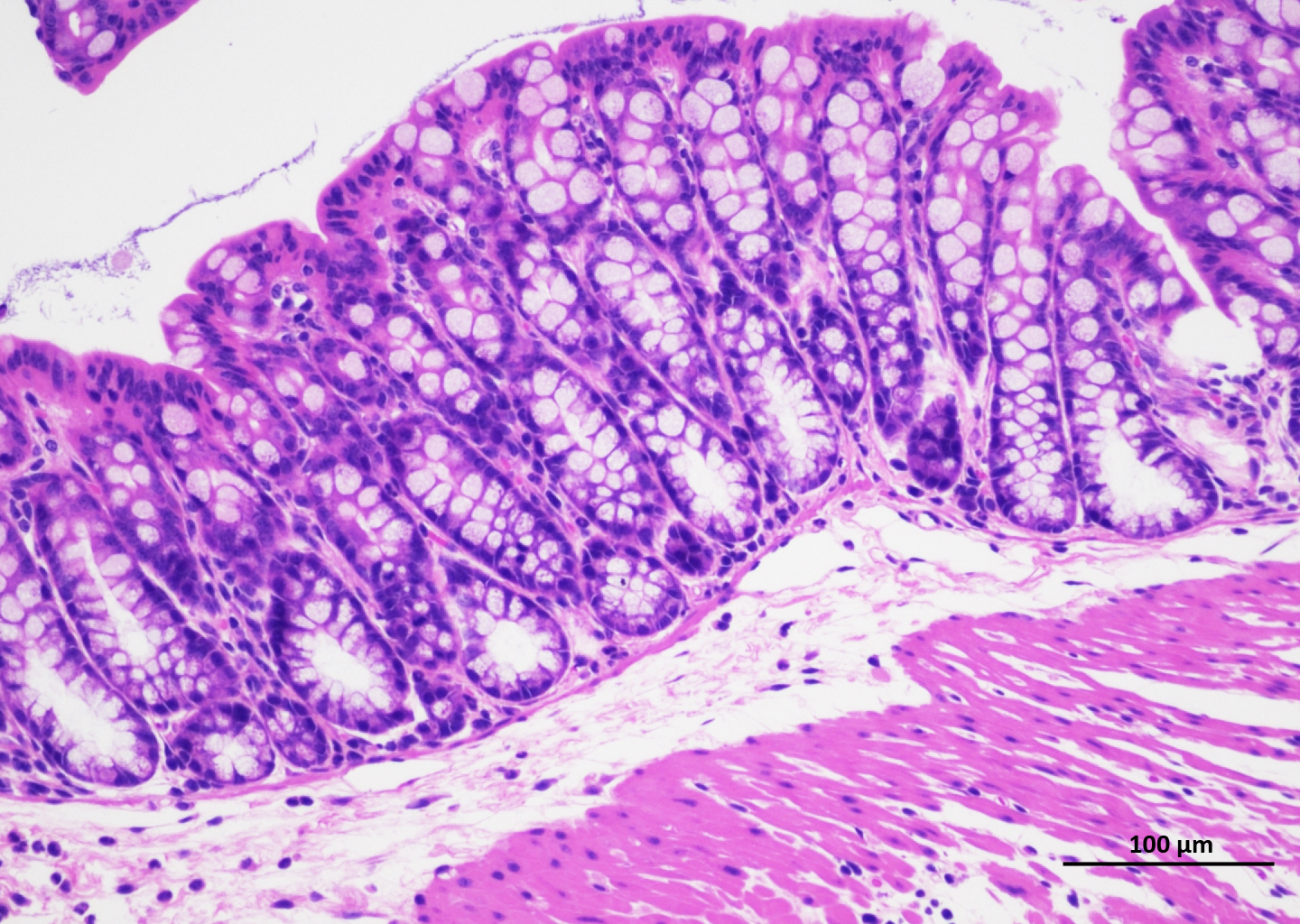

Supplement: Supplementary file 2 [file DataSheet_1.zip › original data/Figure 6/Figure 6A/C.jpg]

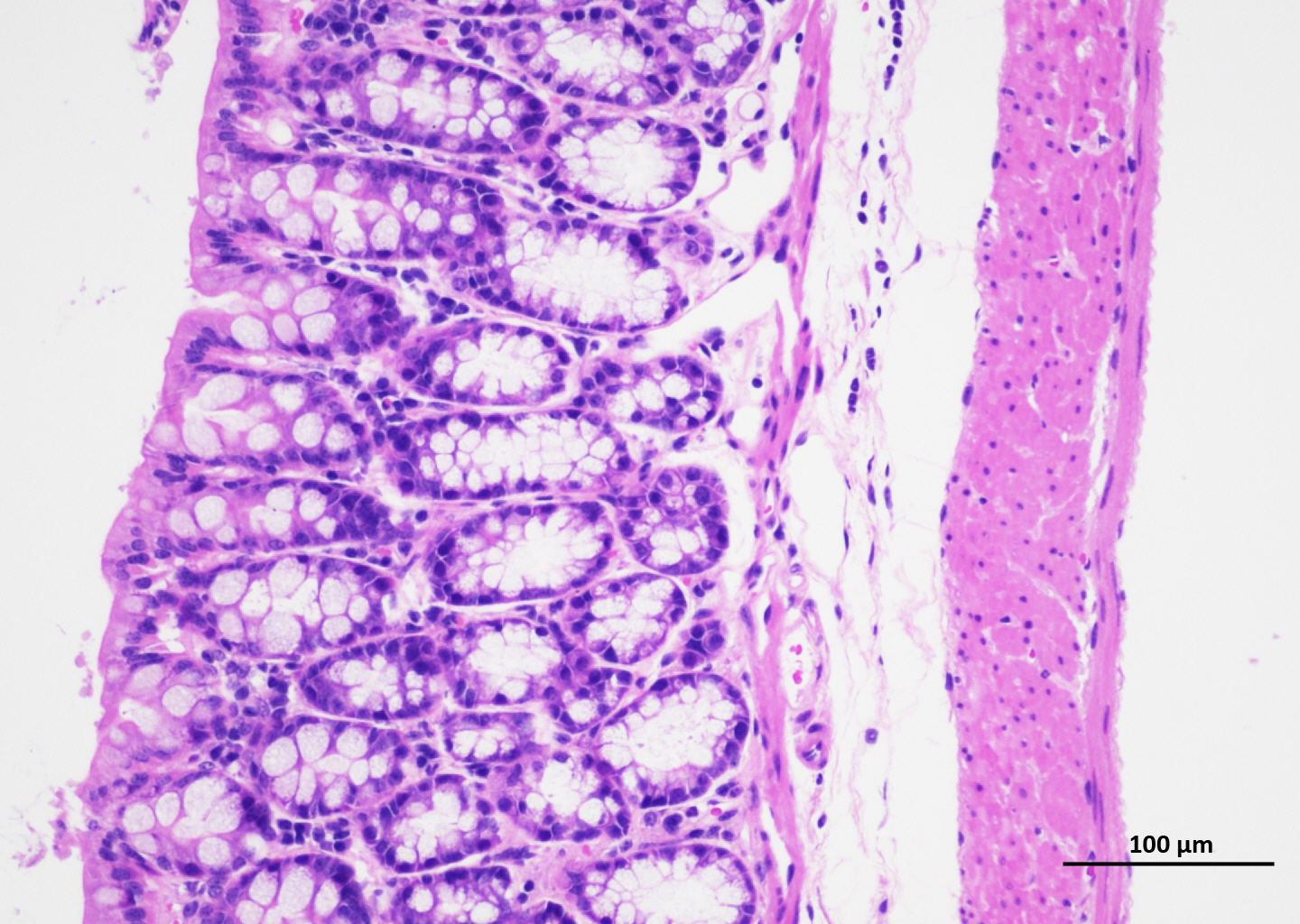

Supplement: Supplementary file 2 [file DataSheet_1.zip › original data/Figure 6/Figure 6A/H.jpg]

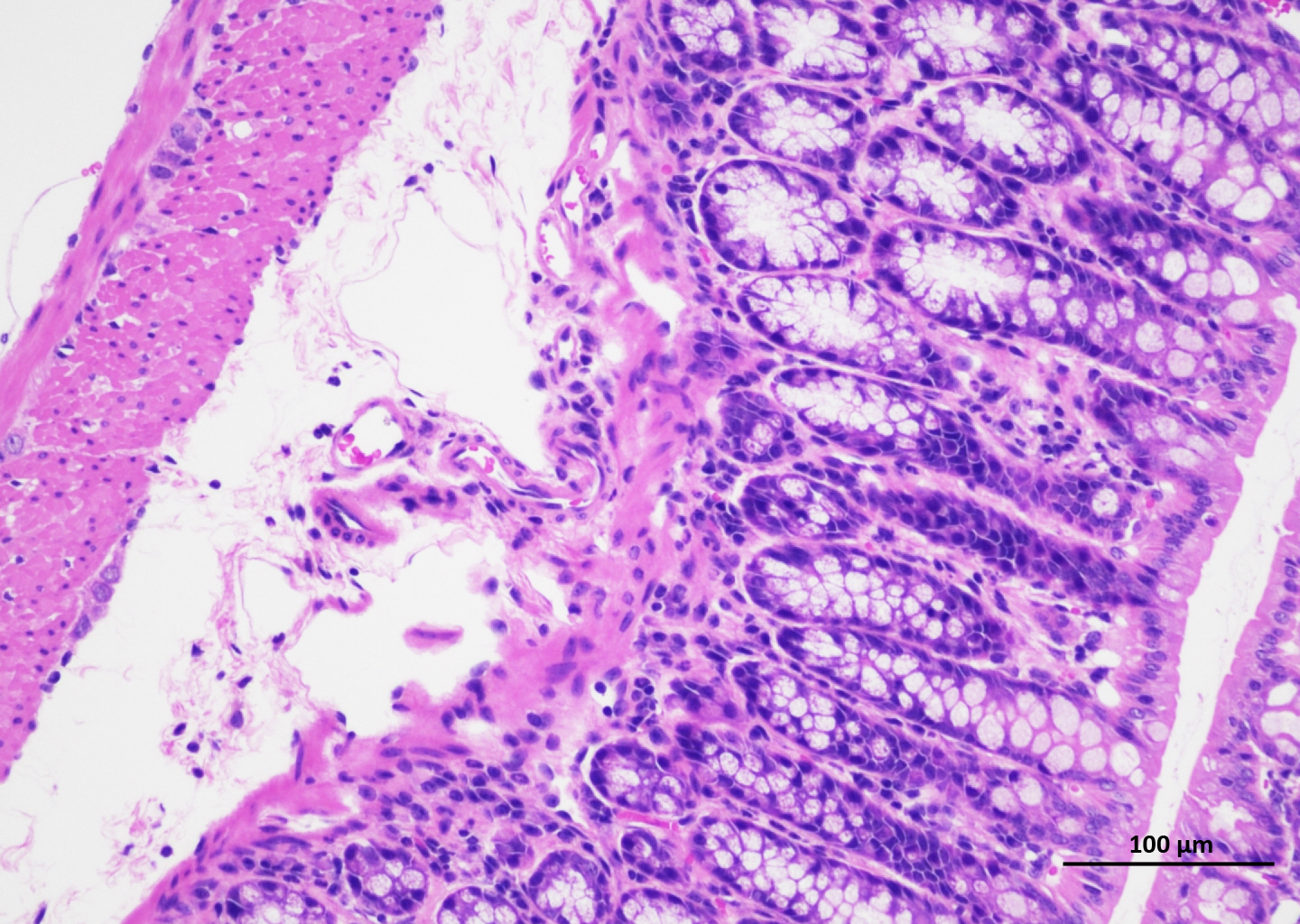

Supplement: Supplementary file 2 [file DataSheet_1.zip › original data/Figure 6/Figure 6A/L.jpg]

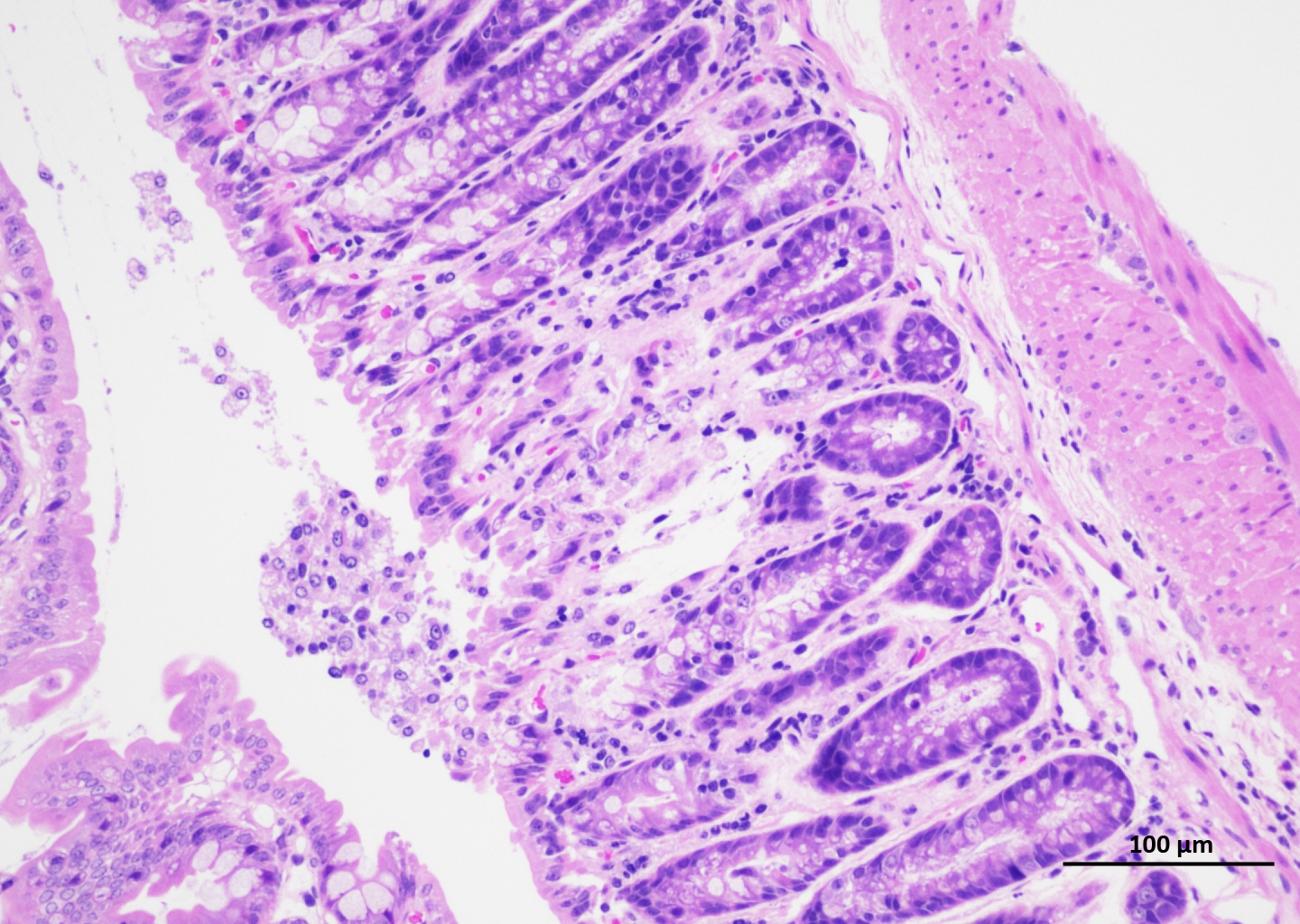

Supplement: Supplementary file 2 [file DataSheet_1.zip › original data/Figure 6/Figure 6A/M.jpg]

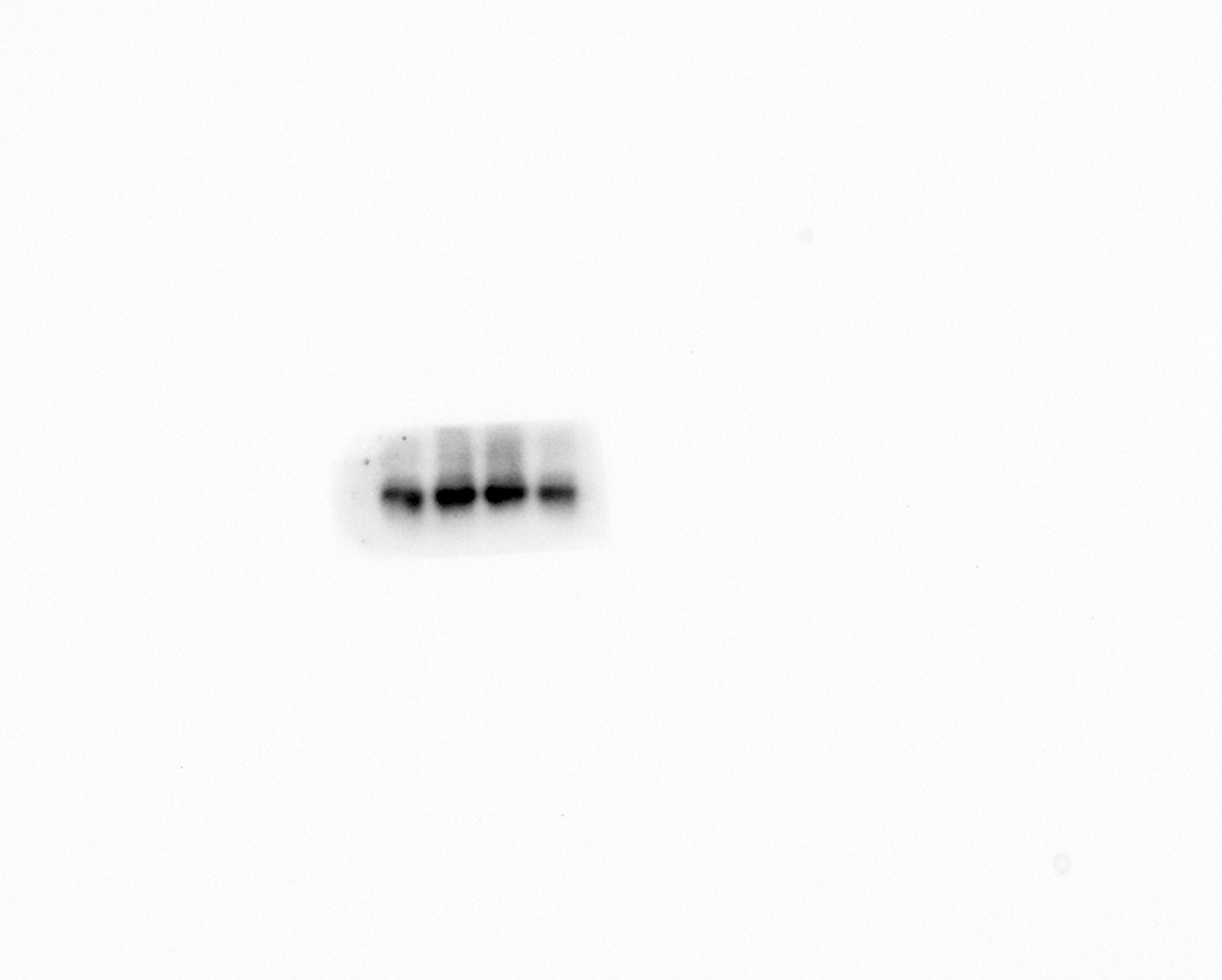

Supplement: Supplementary file 2 [file DataSheet_1.zip › original data/Figure 6/Figure 6B/MYD88.Tif]

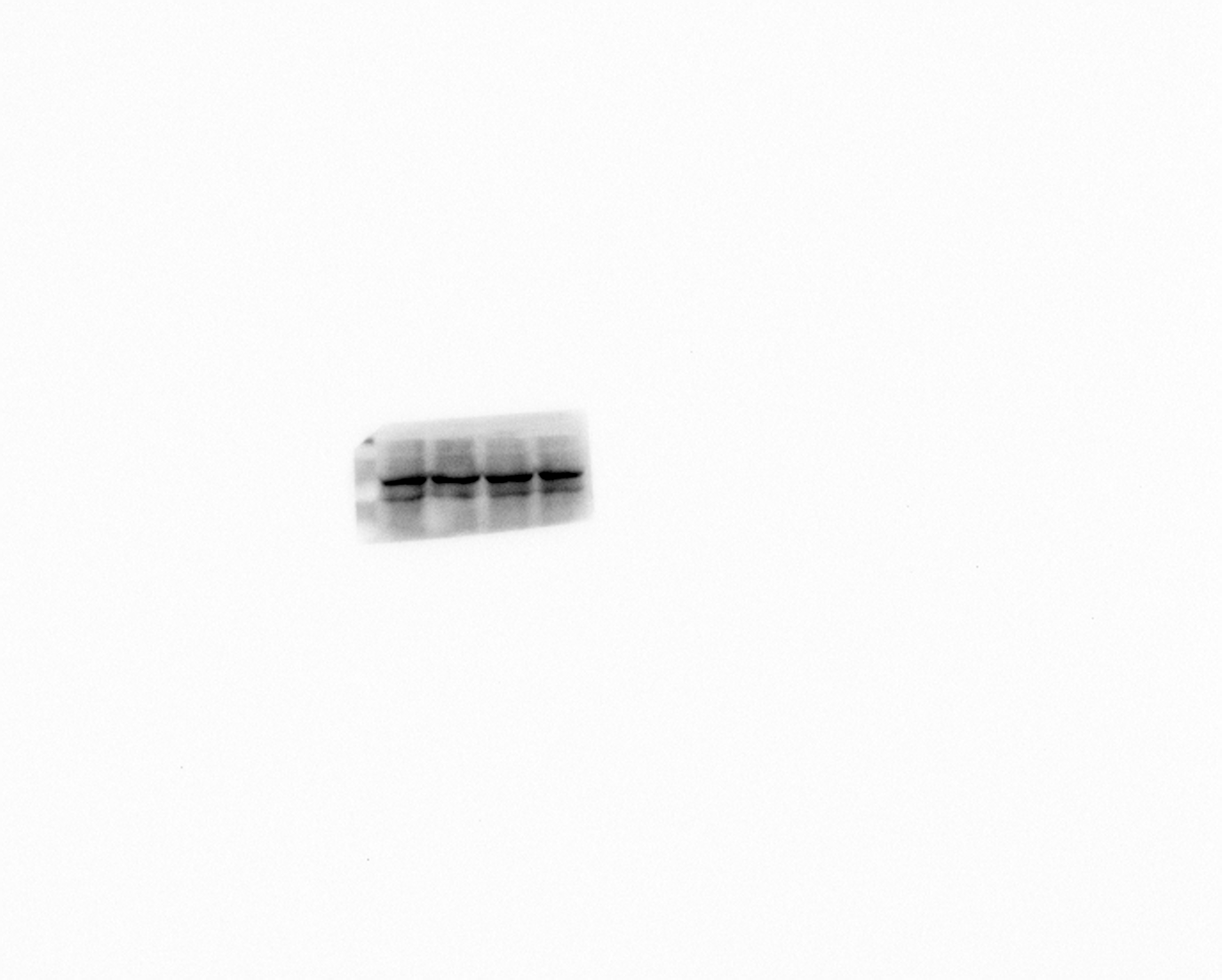

Supplement: Supplementary file 2 [file DataSheet_1.zip › original data/Figure 6/Figure 6B/P65.Tif]

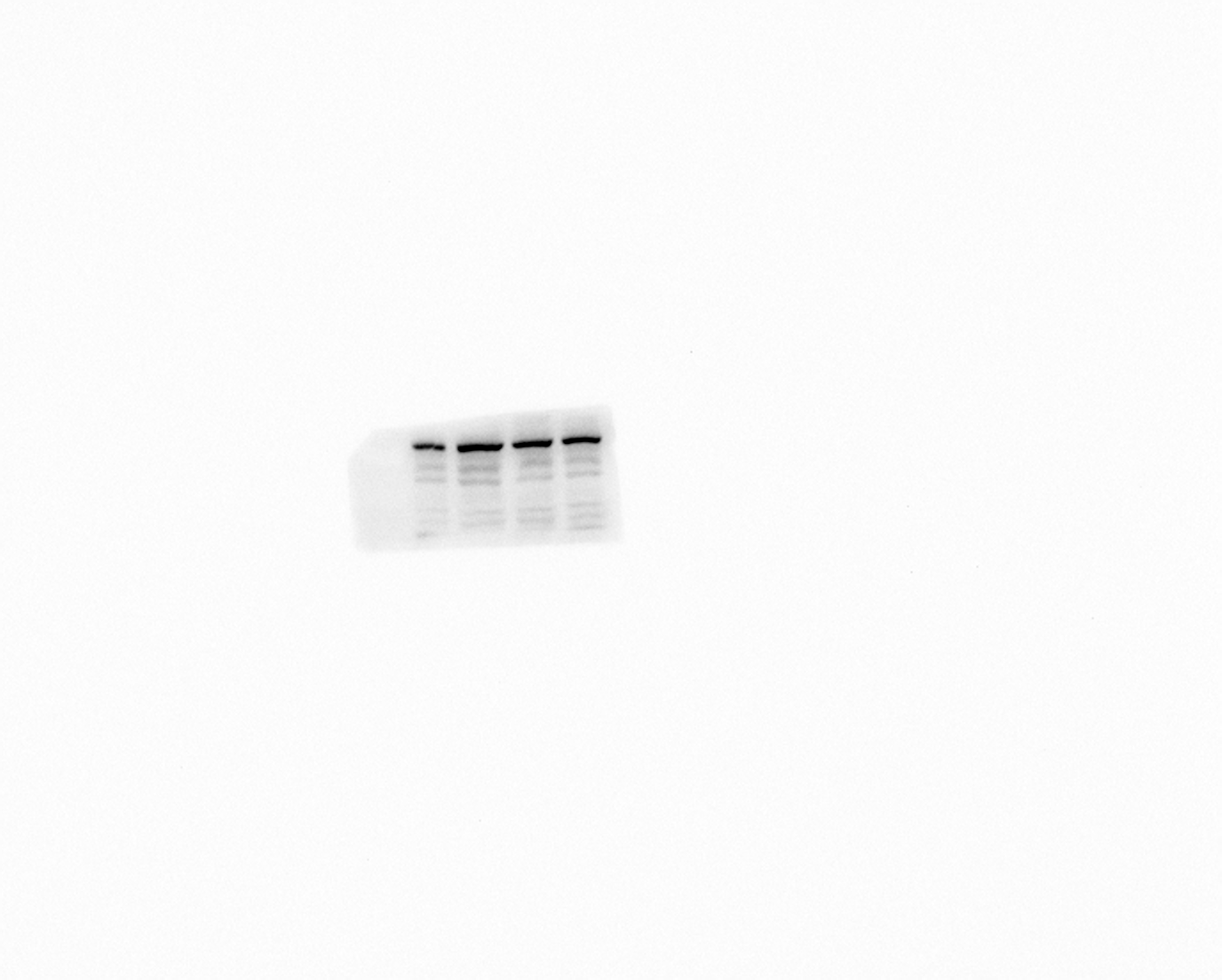

Supplement: Supplementary file 2 [file DataSheet_1.zip › original data/Figure 6/Figure 6B/PP65.Tif]

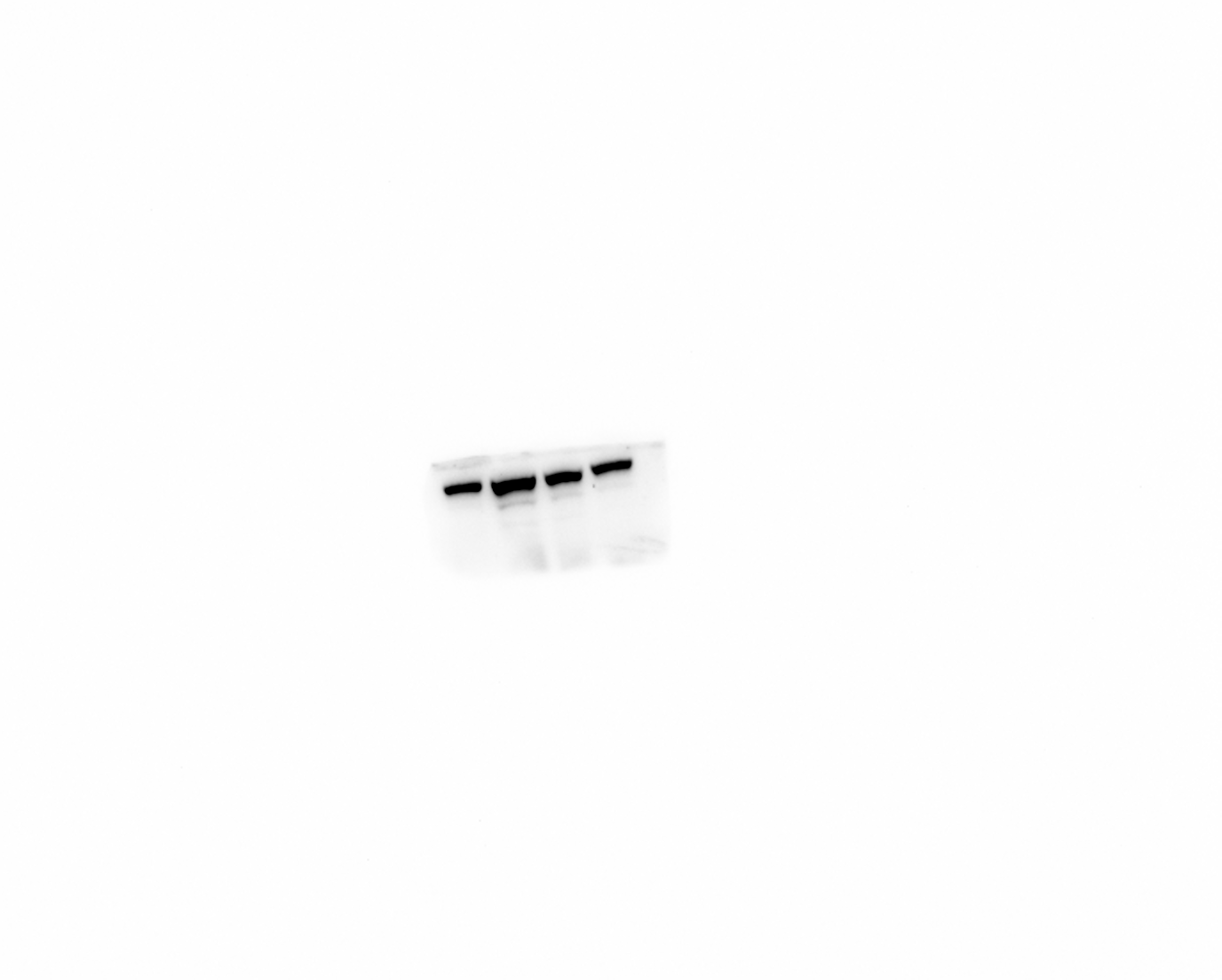

Supplement: Supplementary file 2 [file DataSheet_1.zip › original data/Figure 6/Figure 6B/TLR4.Tif]

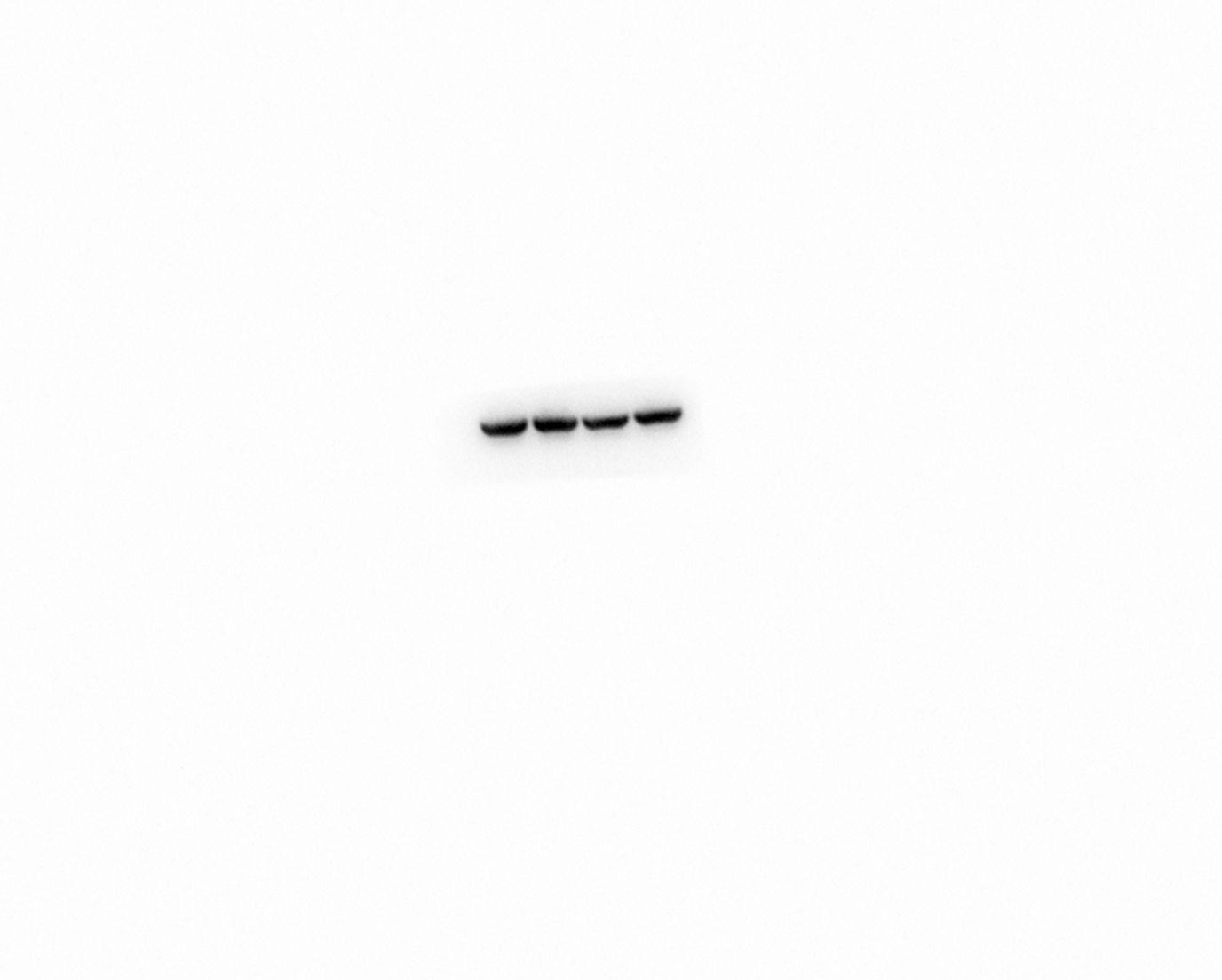

Supplement: Supplementary file 2 [file DataSheet_1.zip › original data/Figure 6/Figure 6B/a┬-ACTIN.Tif]

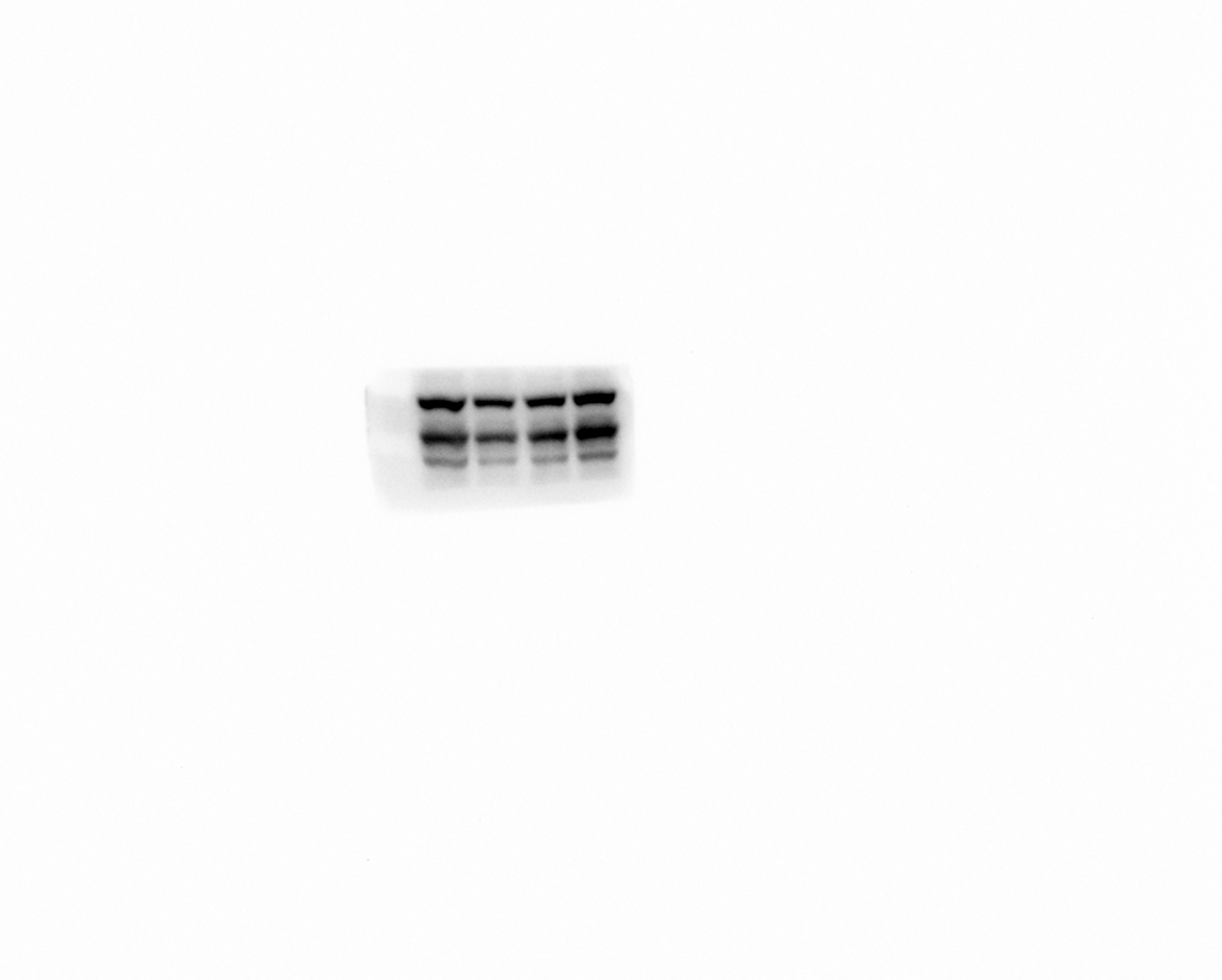

Supplement: Supplementary file 2 [file DataSheet_1.zip › original data/Figure 6/Figure 6C/OCCLUDIN.Tif]

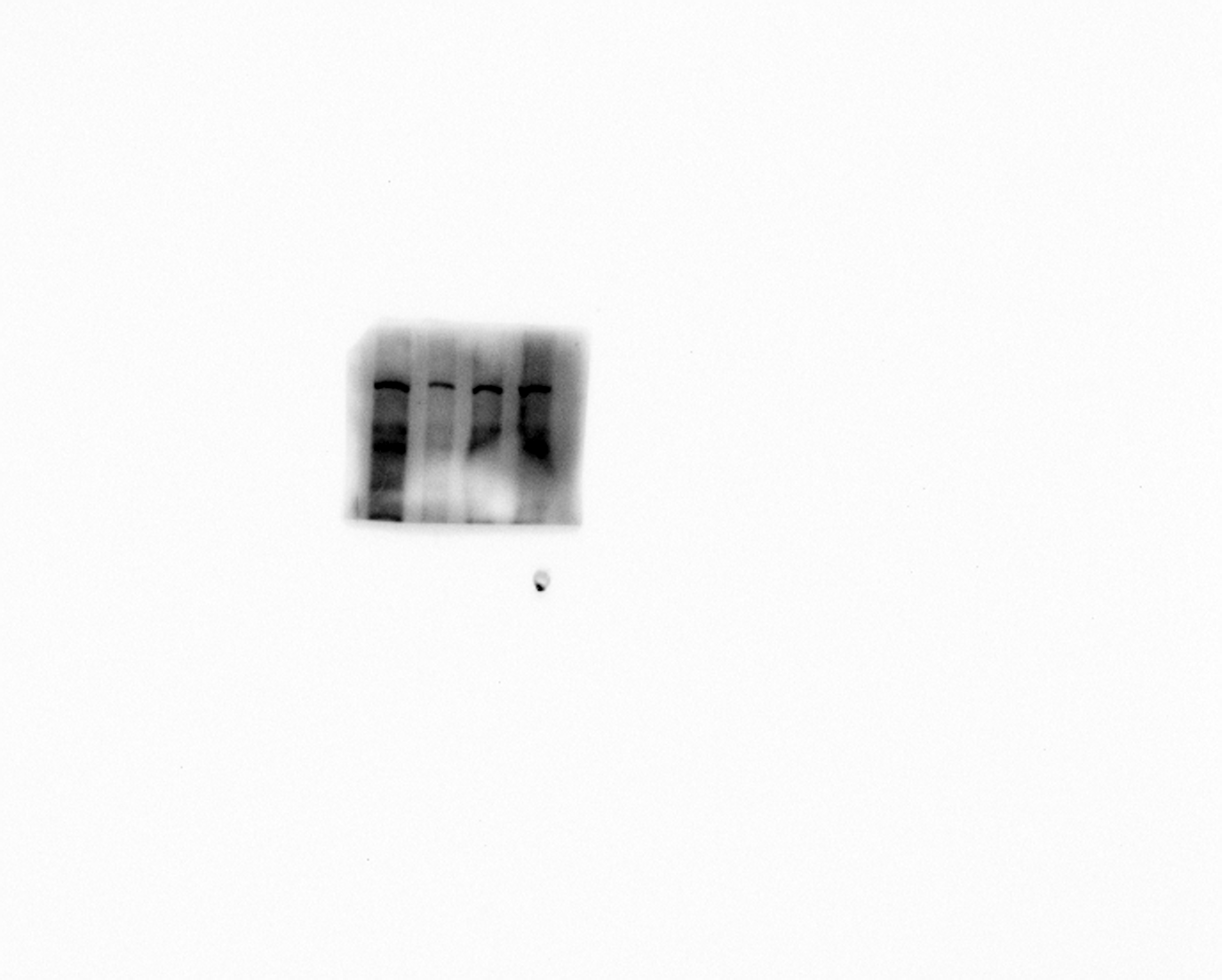

Supplement: Supplementary file 2 [file DataSheet_1.zip › original data/Figure 6/Figure 6C/ZO-1.Tif]

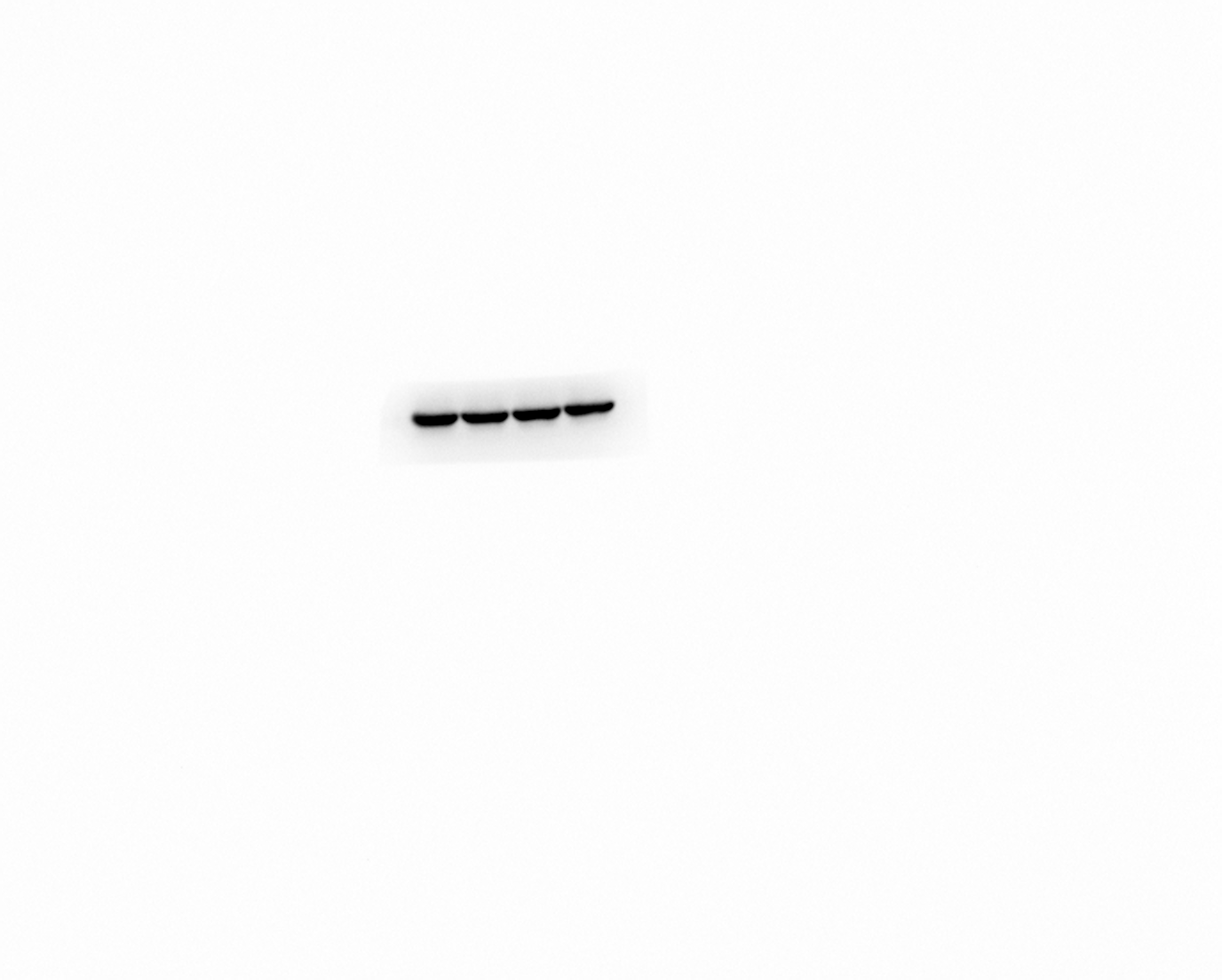

Supplement: Supplementary file 2 [file DataSheet_1.zip › original data/Figure 6/Figure 6C/a┬-ACTIN.Tif]
